# RNAseq revealed the important gene pathways controlling adaptive mechanisms under waterlogged stress in maize

**DOI:** 10.1038/s41598-017-10561-1

**Published:** 2017-09-08

**Authors:** Kanika Arora, Kusuma Kumari Panda, Shikha Mittal, Mallana Gowdra Mallikarjuna, Atmakuri Ramakrishna Rao, Prasanta Kumar Dash, Nepolean Thirunavukkarasu

**Affiliations:** 1 Division of Genetics, Indian Agricultural Research Institute, New Delhi, 110012 India; 2 Amity Institute of Biotechnology, Amity University, Uttar Pradesh Noida, 201 313 India; 3 Centre for Agricultural Bioinformatics, Indian Agricultural Statistics Research Institute, Pusa, Library Avenue, New Delhi, 110 012 India; 4 National Research Centre on Plant Biotechnology, Pusa Campus, New Delhi, 110012 India

## Abstract

Waterlogging causes yield penalty in maize-growing countries of subtropical regions. Transcriptome analysis of the roots of a tolerant inbred HKI1105 using RNA sequencing revealed 21,364 differentially expressed genes (DEGs) under waterlogged stress condition. These 21,364 DEGs are known to regulate important pathways including energy-production, programmed cell death (PCD), aerenchyma formation, and ethylene responsiveness. High up-regulation of *invertase* (49-fold) and *hexokinase* (36-fold) in roots explained the ATP requirement in waterlogging condition. Also, high up-regulation of *expansins* (42-fold), *plant aspartic protease A3* (19-fold), *polygalacturonases* (16-fold), *respiratory burst oxidase homolog* (12-fold), and *hydrolases* (11-fold) explained the PCD of root cortical cells followed by the formation of aerenchyma tissue during waterlogging stress. We hypothesized that the oxygen transfer in waterlogged roots is promoted by a cross-talk of fermentative, metabolic, and glycolytic pathways that generate ATPs for PCD and aerenchyma formation in root cortical cells. SNPs were mapped to the DEGs regulating aerenchyma formation (12), ethylene-responsive factors (11), and glycolysis (4) under stress. RNAseq derived SNPs can be used in selection approaches to breed tolerant hybrids. Overall, this investigation provided significant evidence of genes operating in the adaptive traits such as ethylene production and aerenchyma formation to cope-up the waterlogging stress.

## Introduction

Loss of crop productivity owing to waterlogging threatens the food security in developing countries. Waterlogging condition refers to excess of water in the soil profile, which affects the plant growth and survivability. During longer periods of waterlogging, maize shows delayed growth and decreased yield^1^. The most impeding effect of waterlogging on plant growth is the arrested oxygen supply to the root system. Such conditions deter the root porosity and oxygen movement in waterlogging sensitive species^2^. However, adaptive traits such as enhanced shoot elongation, shoot porosity, and adventitious root porosity are linked to enhanced tolerance in waterlogged plants^2, 3^.

Aerenchyma formation is one of the most important tolerance mechanisms in waterlogged maize. Many wetland plants form gas spaces between cells (aerenchyma) in order to enhance the oxygen uptake in roots under waterlogging conditions as an escape strategy^4, 5^. Ethylene-^6, 7^ and ROS-dependent signaling enhances the programmed cell death (PCD) in root cortex^8^. PCD includes various signal transductions which involve Ca^++^^9^and protein kinases like mitogen-activated protein kinases (MAPK)^10^ that trigger transcriptional regulators. These functional pathways facilitate the degradation of cell wall involving proteases^11^ and nucleases^12^ allowing the formation of gas-filled cavities called as aerenchymous tissue.

Along with the induction of PCD and aerenchyma formation, ethylene and its transcriptional regulators such as ethylene response factor (ERF) promote an N-end rule pathway in hypoxic conditions^13^. Group VII of ERF transcription factors are MC (Methionine-Cysteine)-initiating proteins that are converted to active ERFs after the removal of methionine^14^. These active ERFs are degraded under normal oxygen and induce hypoxic core responsive genes that regulate fermentation, sucrose metabolism, ethylene production, cell death-related, glycolysis, and ROS production under low oxygen conditions^13^. Tolerance mechanisms have an accumulative effect towards plant survival under stressed conditions.

During waterlogging, plants maintain the ATP production through substrate-level phosphorylation^15, 16^ in order to supply ATP to energy-consuming formation of aerenchyma in root cortical cells^17^. Lactate and ethanol fermentation is one of the energy-producing mechanisms that are stimulated under low oxygen conditions^18^. These fermentation pathways provide NAD^+^ to maintain glycolysis which in turn increases the ATP generation. However, glycolysis is preserved by increased glucose levels through sucrose metabolism. As a result, the ATP produced is consumed by the development of gas films to enhance the oxygen uptake in waterlogging conditions^17^.

The genetic control of tolerance variation and understanding the underlying genes and regulating pathways can be comprehended with genome-wide studies. RNA sequencing is one of the trending techniques that allow the study of expression levels of all mRNAs in a transcriptome. In our study, we explored functional pathways and the regulating genes expressed in waterlogged maize. Our study identified novel tolerance mechanisms which could be ultimately translated to breed tolerant maize genotypes to improve the productivity under waterlogged agriculture systems.

## Results

### Differentially Expressed Genes (DEGs) in Waterlogged Roots

We generated whole genome transcripts from the root tissue of a tolerant line (HKI 1105) under non-stress and waterlogging stress conditions. RNA samples were then sequenced on Illumina flow cells that generated 44 and 47 million sequencing reads. These reads were then mapped onto the reference B73 genome and classified into two groups: reads mapped as an intact pair and reads mapped in broken pairs. Both datasets yielded at least 23 million reads mapped exactly onto the reference genome and at least 5 million reads mapped in broken pairs (Supplementary Table S1).

Each RNA-sequencing dataset was maintained with two biological and two technical replications where all reads were pooled as paired sequencing reads after mapping. These paired sequencing reads were counted in the form of fragments (15,953,086) out of which 60.9% were unique and 39.1% were non-specific fragments in waterlogged root sample where the counted fragments mapping the transcripts were translated to raw count-based and normalized expression values. On the other hand, the total paired sequencing reads counted to 11,705,441 fragments in non-stress root sample, out of which 57.4% were unique and 42.6% were non-specific. Proportion-based statistical test (Baggerley *et al*.’s test)^19^ was applied to both non-stress and stress samples where the expression levels are compared at the proportion level and the data is corrected for sample size. Raw count-based expression values counted in the form of reads were normalized to account for biases that occur between sequencing runs and sequencing platforms. The normalized expression values counted for each gene included reads per million (RPM) and reads per kilobase million (RPKM). RPKM values obtained in both samples were transformed to fold changes that explained the differences in expression of stress over non-stress sample. We used a threshold level of 1 RPKM in each sample in order to designate a gene as expressed, accounted to 12,633 maize gene models in non-stress and 13,872 in stress sample (Supplementary Table S1). A gene expressed at a fold change (>1) between stress and non-stress was considered as a differentially expressed gene (DEG) in waterlogging condition. A total number of 21,364 DEGs encoded transcripts each above 150 bp concluding 4.6% coverage of the maize genome in waterlogged root sample. Out of these 21,364 DEGs, 30% genes were annotated which accounted for 21 Mb of the maize genome. In addition, these 21,364 DEGs included 13,771 up-regulated and 7,593 down-regulated genes in stress condition. The maximum up-regulation was identified for *invertase* (49-fold) followed by the *expansins* (42-fold) and *hexokinase* (36-fold); and maximum down-regulation was identified for *F-box ubiquitin ligases* (64-fold) followed by the *RING finger ubiquitin ligases* (44-fold and 34-fold) in waterlogging conditions (Supplementary Tables S4–S7). These DEGs regulated different stress tolerant pathways such as energy-production (71), programmed cell death (PCD) (29), aerenchyma formation (53), and ethylene-responsive pathways (667). DEGs identified in the waterlogged roots were validated through qRT-PCR assay. The expression pattern of selected DEGs in qRT PCR assay demonstrated good consistency with the RNA-seq expression analysis (Supplementary Fig. 1). The genes *hexokinase*, *invertase*, *RBOH*, *PASPA3*, *expansins*, and *ubiquitin ligases* were up-regulated; and *LDH*, *MT* and *polygalacturonases* were down-regulated in stress sample.

### Functional Annotation and Classification

**Figure 1:**
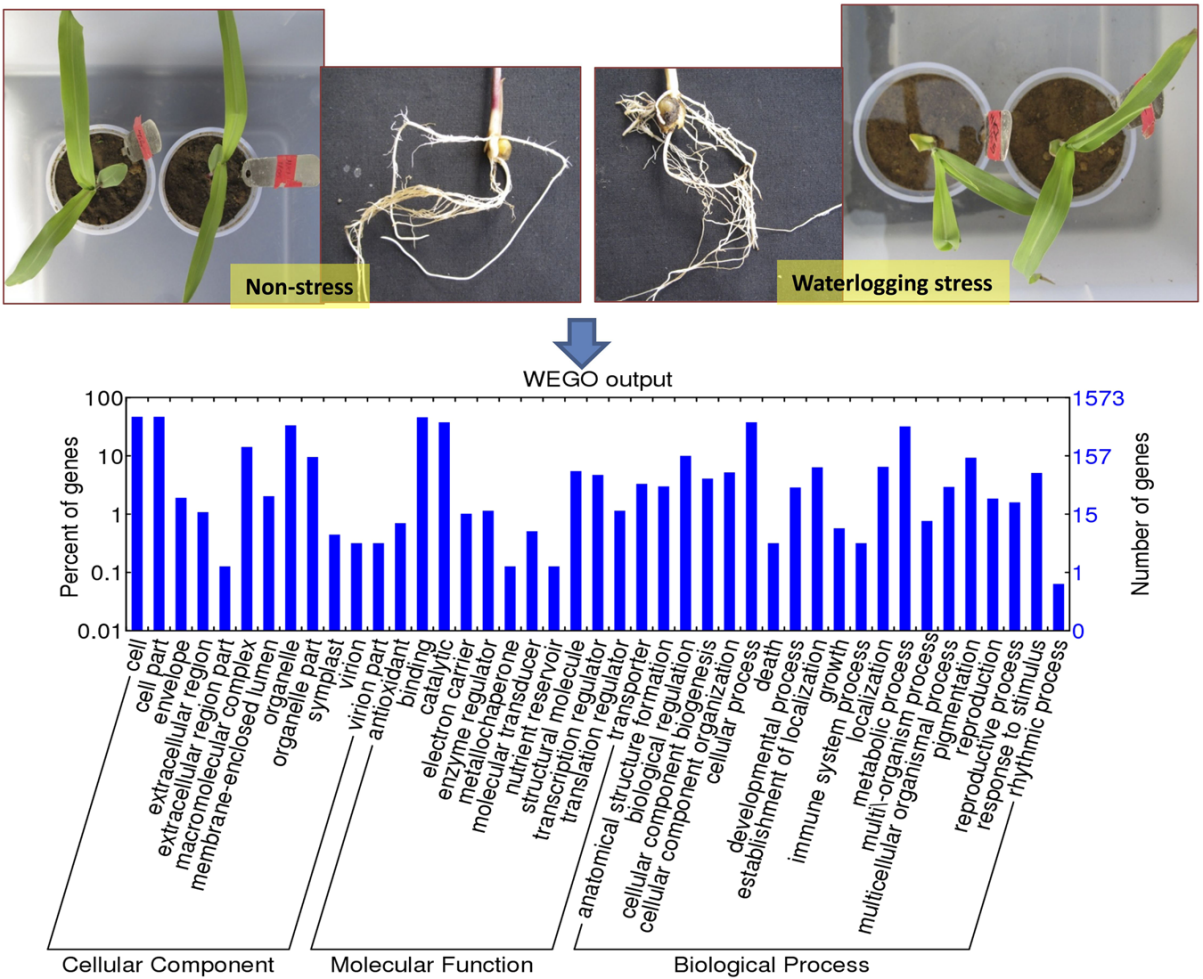
Representation of differentially expressed genes during waterlogging stress in selected Gene ontology (GO) categories in maize. Differentially expressed genes were annotated according to GO categories cellular component, molecular function, and biological process. Number of genes represent the differentially expressed genes in waterlogging tolerant genotype HKI 1105.

Top BLAST hits of the transcripts sequenced were assigned GO terms, namely, cellular component, molecular function, and biological process. Out of total RNAs sequenced, 1573 DEGs were categorized to GO terms. Of 1573 transcripts, 60–70% transcripts were annotated to seven categories: GO cellular component category “cell”, “cell part”, and “organelle”; GO molecular function category “catalytic binding” and “catalytic activity”; and GO biological processed category “cellular process” and “metabolic process” (Fig. 1). Transcripts sequenced in non-stress sample were annotated to various metabolic pathways where maximum transcripts were annotated to TCA cycle (445) followed by lipid metabolism (288), amino acid metabolism (216), and cell wall metabolism (213). In addition, transcripts sequenced in the same sample were also annotated to regulatory pathways where maximum transcripts were annotated to transcription factor (1539) followed by protein degradation (1154). Transcripts sequenced in stress sample were annotated to various metabolic pathways where maximum transcripts were annotated to TCA cycle (446) followed by lipid metabolism (288), amino acid metabolism (216), and cell wall metabolism (213). In addition, transcripts sequenced in the same sample were also annotated to regulatory pathways where maximum transcripts were annotated to transcription factor (1540) followed by protein degradation (1154).

It was studied that similar number of transcripts sequenced in non-stress and stress samples were annotated to metabolic and regulatory pathways. Contrastingly, the genes annotated to TCA (EF517601.1_FGT015) and transcription factor (EF517601.1_FGT010) were sequenced only in the stress sample. The former transcript was up-regulated at 1-fold and the latter transcript was down-regulated at 3.6-fold in stress conditions. In addition, these transcripts were annotated to uncharacterized proteins in maize.

### Polymorphism in the Expressed Sequences

**Figure 2:**
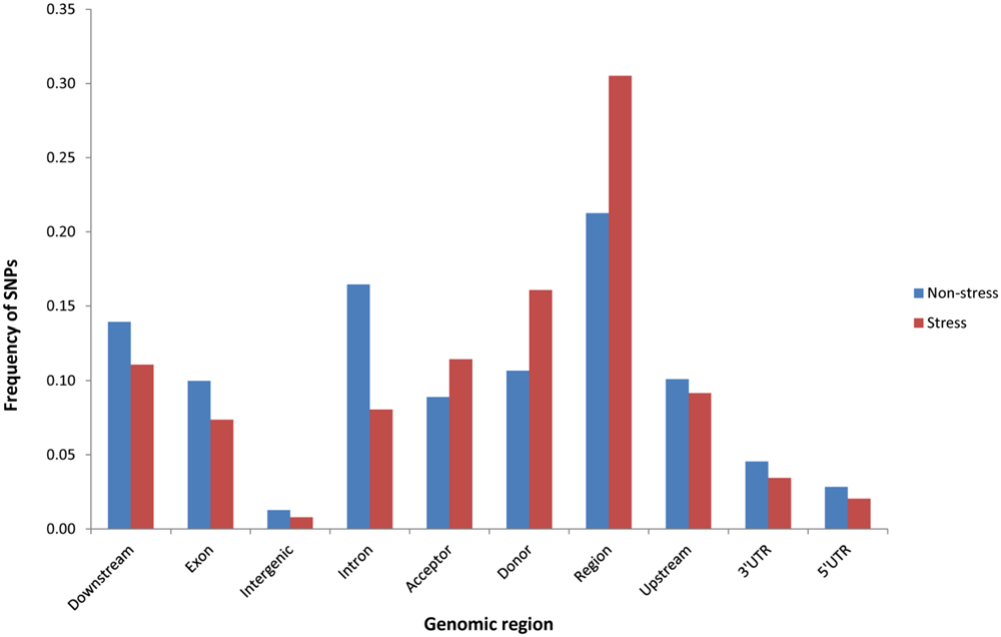
Frequency of SNPs mapped in genomic regions in waterlogging tolerant genotype (HKI 1105) of maize. SNPs were mapped differentially in various genomic regions of non-stress and stress samples. X-axis represents the genomic regions in which SNPs were mapped and y-axis represents SNP frequency.

RNA sequencing of each of the non-stress and stress root sample resulted to 13 Gb sequenced data. On an average, one SNP was called every 52 kb in stress sample and 53 kb in non-stress sample. The expressed transcripts of each sample were compared with B73 maize genome to find out the SNPs (Table 1). Our study revealed 40,166 and 41,283 polymorphisms (SNPs and INDELs) in non-stress and stress samples (Table 1). In non-stress sample, 40,166 polymorphisms represented 8,688 synonymous and 17,930 non-synonymous SNPs. In stress sample, 41,283 polymorphisms represented 4,628 synonymous and 5,763 non-synonymous SNPs. SNPs and INDELs were mapped to 30% of the total reads (1,66,235 read counts) in splicing site regions (Fig. 2, Supplementary Table S2). About 20% of the total reads (1,10,267 read counts) were mapped in downstream and upstream regions of SNPs and INDELs (Fig. 2, Supplementary Table S2). Moreover, SNPs and INDELs were also mapped to exonic regions that concluded 7.4% of the total reads (40,133 read counts) (Fig. 2, Supplementary Table S2).

**Table 1:** Summary of polymorphisms identified in the non-stress and stress samples.

Category	Non-stress	Stress
Total number of polymorphisms	40,166	41,283
SNP	16,599	8,415
INS	21,395	30,572
DEL	2,172	2,296
Transitions	30,574	14,470
Transversions	22,783	11,714
Ts/Tv ratio	1.342	1.2353

Amino acid substitutions were identified in SNPs mapped in non-stress and stress samples with reference to B73 maize genome. In non-stress sample, glycine to valine (768), alanine to serine (695) followed by valine to alanine (632), and threonine to alanine (583) (Supplementary Table S3A) were the major amino acid substitutions whereas in stress samples, it was found that lysine to asparagine (499) followed by valine to alanine (418), and tyrosine to leucine (401) (Supplementary Table S3B) were the major amino acid substitutions.

In addition, polymorphism due to transitions (Ts) and transversions (Tv) were also estimated in each sample. In non-stress, Ts and Tv amounted to 30,574 and 22,783 respectively while in stress, Ts and Tv counted to 14,470 and 11,714 respectively.

### Stress Tolerant Pathways

Genes expressed differentially during waterlogging stress regulate various stress tolerance pathways. Energy in the form of ATP is required by cells to achieve tolerance to waterlogging stress in plants. In this study, the regulating genes to energy-production pathways were differentially expressed in maize during waterlogging stress. Waterlogging tolerance includes aerenchyma formation through PCD of root cortical cells in plants. The gene network to PCD and aerenchyma formation was explained explicitly in genes expressed differentially in waterlogging stress. Such changes in plants are regulated by ethylene through ethylene-responsive pathways. Ethylene acts as a stress hormone in plants that induces the transcription of various stress-responsive genes. These pathways counteract the tolerance of maize to waterlogging stress through a complex network of genes.

#### Energy-Production Pathways

Energy-production pathways include fermentation of pyruvate to ethanol and metabolism of sucrose to glucose which is utilized by glycolysis for ATP production. Metabolism of sucrose to glucose is catalyzed by invertase and sucrose synthase, where both of them were up-regulated and differentially expressed in waterlogged roots (Supplementary Table S4). Here the maximum expression was noted at 49-fold change in an *invertase* gene (GRMZM2G119689) and 7-fold change in a *sucrose synthase* gene (GRMZM2G152908) in stressed condition. Glucose is converted to pyruvate which involves a series of glycolytic enzymes of which nine encoding DEGs were up-regulated under waterlogging condition (Supplementary Table S4). The first step of glycolysis, addition of phosphate to glucose which forms glucose-6-phosphate, is catalyzed by hexokinase, of which the maximum encoding DEGs (7) were up-regulated in stressed condition. The highest fold-change was noted up to 36-fold for GRMZM2G046686, denoting hexokinase. Isomerization of glucose-6-phosphate to fructose-6-phosphate was catalyzed by phosphoglucose isomerase, accounting for two DEGs, GRMZM2G065083 up-regulated at 1.2-fold and GRMZM2G140614 up-regulated at 2-fold in waterlogged roots. Fructose-6-phosphate is converted to fructose-1,6-bisphosphate by the action of phosphofructokinase, of which six DEGs were up-regulated and two were down-regulated in stressed condition. Phosphofructokinase was observed for a maximum of 7-fold (GRMZM5G879882). Glycolysis involves an equilibration step which maintains the glucose level in cytosol: fructose-6-bisphosphate is converted into two glycolytic products, glyceraldehyde-3-phosphate and dihydroxyacetone phosphate by the action of aldolase enzyme. Aldolase encodinggenes were up-regulated and down-regulated for two gene models each, where the highest differential expression (1.6-fold) was observed for GRMZM2G057823 in stressed condition. Triose phosphate isomerase is the fifth glycolytic enzyme, of which GRMZM2G030784 was differentially expressed with upregulation at 1.2-fold in waterlogged roots. Two dehydrogenases; glyceraldehyde-3-phosphate-dehydrogenase (GAPDH) and 3-phosphoglycerate dehydrogenase (PHGDH) encoding DEGs were also up-regulated where GRMZM2G051004 (GAPDH) and GRMZM2G009323 (PHGDH) were expressed above 2.6-fold in stressed condition. Phosphoglycerate kinase (PGK) is another glycolytic enzyme, whose four encoding DEGs were up-regulated where GRMZM2G003724 was expressed at 8.8-fold in stressed roots. The last step of glycolysis of phosphoenolpyruvate to pyruvate was catalyzed by enolase enzyme, of which one encoding DEG GRMZM2G034848 was up-regulated at 1.7-fold and other GRMZM2G481529 was down-regulated in stressed condition. The final product of glycolysis pyruvate is fermented into lactate and ethanol *via* two different pathways: lactate fermentation catalyzed by lactate dehydrogenase (LDH) and; ethanol fermentation catalyzed by pyruvate decarboxylase (PDC) and alcohol dehydrogenase (ADH). Amongst the three encoding genes, *ADH* was differentially expressed at a highest level of 7.6-fold in waterlogging conditions (GRMZM2G152981) followed by *PDC*.

#### *Programmed Cell Death* (*PCD*) *and Aerenchyma Formation*

Programmed cell death (PCD) and formation of an aerenchymous tissue allows gaseous exchange between roots to the other parts of the plant. Calcium (Ca^2+^) signaling is an important part of the PCD signaling which involves phosphorylation of proteins by protein kinases such as mitogen-activated protein kinases (MAPK) that further induce other transcriptional regulators. In our study, eight DEGs encoding *MAPK* were up-regulated and other three were down-regulated (Supplementary Table S5) in waterlogged roots. PCD is an ethylene-responsive mechanism in plants where ethylene biosynthesis and accumulation promotes cell death and lysis in root cortical cells. In the cortex portion of root, respiratory burst oxidase homolog (RBOH) catalyzes the conversion of oxygen molecule into reactive oxygen species (ROS) which is scavenged by metallothionein (MT) in normal conditions. Our study found six DEGs up-regulated for *RBOH* (GRMZM2G034896 highly expressed at 12.1-fold) and two down-regulated in waterlogging conditions (Supplementary Table S5). In waterlogging conditions, low oxygen persists where MT is present in low quantities, as a result of which ROS is not scavenged that allows cell death and its lysis in root cortical cells. Two DEGs were down-regulated for MT upto 7-fold (Supplementary Table S5) in stressed condition.PCD includes the lysis of cells by the degradation of cell wall by nucleases and proteases. Amongst the nucleases one differentially expressed gene encoding bifunctional nuclease 1 (BFN1) was up-regulated at 3.2-fold (Supplementary Table S5) in stressed condition. In case of proteases, five genes encoding *plant aspartic protease A3* (*PASPA3*) were up-regulated with highest differential expression of 18.7-fold (GRMZM2G036134) and one was down-regulated (Supplementary Table S5) in waterlogged roots. Cell wall modification promotes the formation of a gaseous tissue called aerenchyma. Such changes in the cell wall in waterlogging conditions were observed by the differential expression of four cell wall modification enzymes encoding genes in waterlogged roots (Supplementary Table S6). About 18 DEGs encoding expansins were up-regulated (highest expression of GRMZM2G094523 at 41.8-fold) and four were down-regulated in stressed condition. One DEG GRMZM2G412207 encoding pectin lyases was up-regulated with 1.2-fold and three were down-regulated in stressed condition. In total 16 DEGs accounting for polygalacturonases were up-regulated (highest expression of GRMZM2G467435 at 16.1-fold) and three were down-regulated. Five DEGs for *xyloglucan endo-transglycosylasesor hydrolases* (*XTH*) were up-regulated where GRMZM2G060837 was highly expressed at 10.7-fold and three were down-regulated in waterlogged condition.

#### *Ethylene*-*Responsive Pathways*

Ethylene plays an important hormone as it induces several tolerance mechanisms in waterlogging conditions. Ethylene is synthesized by ACC synthase (ACS) of which two encoding genes were differentially expressed and up-regulated (GRMZM2G377341 highly expressed at 1.2-fold; AC197672.3 highly expressed at 2.6-fold) in waterlogged roots (Supplementary Table S7). Group VII Ethylene Response Factor (ERFVII) transcription factors are degraded in normoxia through N-end rule pathway and stable in hypoxic conditions. Six N-end rule pathway substrates were differentially expressed in waterlogged roots in this study. ERFVII is the first substrate with a methionine-cytosine-initiating sequence, of which four encoding genes were up-regulated with high differential expression (GRMZM2G018398) at 2.8-fold and four were down-regulated (Supplementary Table S7) in stressed condition. Methionine is cleaved from ERFVII by the action of methionine aminopeptidase (MetAP) that accounted for four encoding up-regulated genes with maximum (GRMZM2G068982) differential expression at 3.5-fold and two encoding down-regulated genes (Supplementary Table S7) in waterlogged condition. This form of ERFVII is considered as stable as it induces hypoxia-responsive core genes^20^. ERFVII is degraded by a series of events catalyzed by three enzymes: Arginyl-tRNA-protein transferase (ATE), E3 Ubiquitin ligases, and 26 S proteasomes. *ATE* gene was noted for low differential expression (1-fold) in waterlogging condition. E3 Ubiquitin ligases and 26 S proteasomes are enzymes that cleave unstable proteins in a genome and in this study, varying forms of E3 ubiquitin ligases and 26 S proteasomes were differentially expressed in waterlogged roots (Supplementary Table S7).

## Discussion

### Genes Expressed in Adaptive Waterlogging Tolerance Pathways

We investigated the large-scale changes in transcript abundance in waterlogged roots of maize with the assistance of high-throughput RNA-Sequencing of the cDNA library prepared from their root samples. Environmental stimuli accumulate changes in transcript abundance of many protein-encoding genes and transcription factors^21, 22^ implicated in many regulatory mechanisms under waterlogging conditions. Our study aimed at intensifying the differential expression profiling of regulatory genes responsible for energy-production pathways, programmed cell death (PCD), aerenchyma formation, and ethylene-responsive pathways, where the entire regulatory network allows the development of tolerance levels in stressed conditions.

#### Energy-Production Pathways

**Figure 3:**
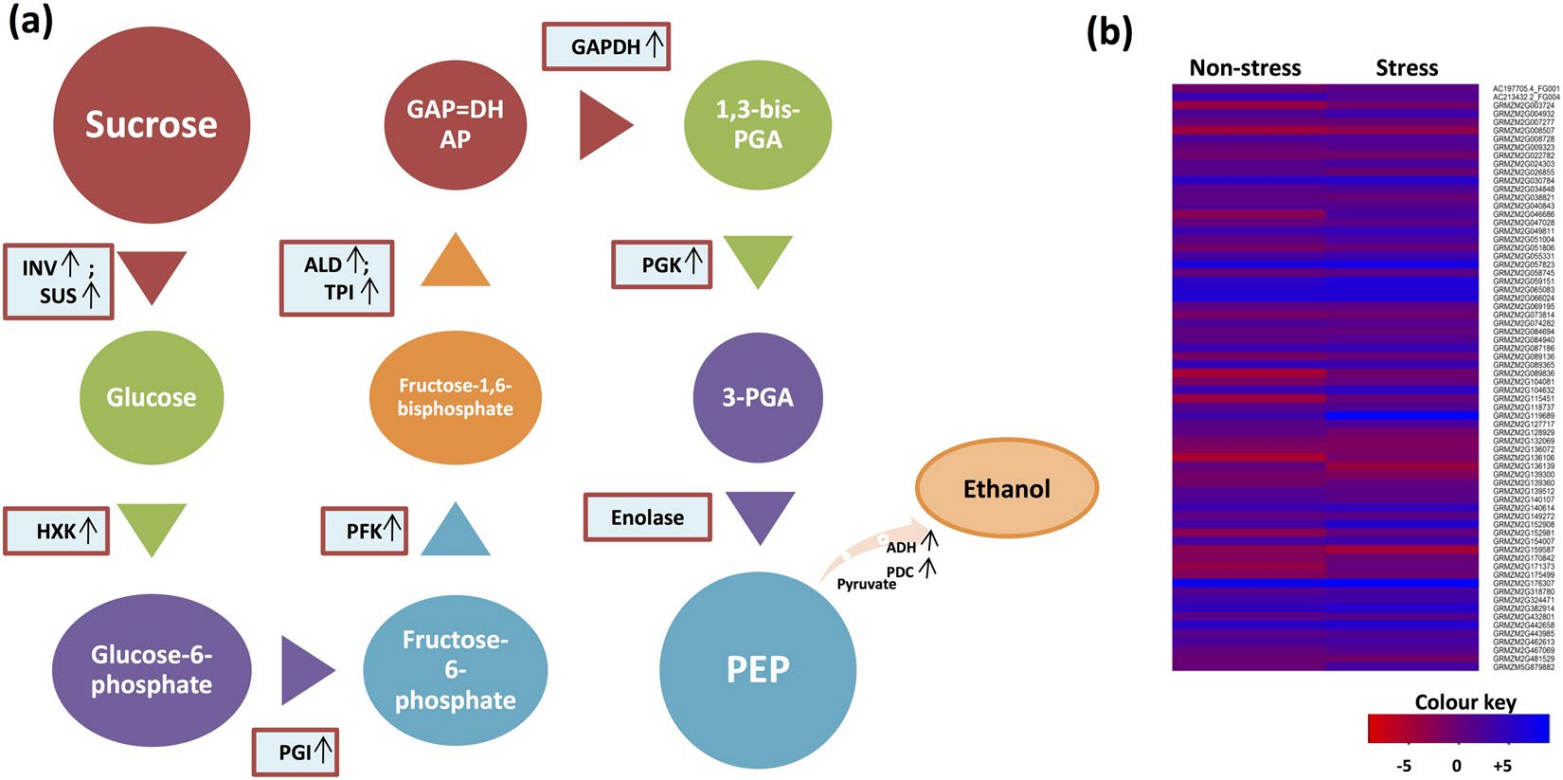
Energy-production pathways regulated in waterlogging tolerant genotype under stressed condition. **(a)** Energy-production pathways regulated during waterlogging stress. Here arrows denote the expression level of waterlogging responsive genes in maize roots. Expression level marked here includes greater than 2 fold change. Refer the paper for abbreviations. **(b)** Heatmap of energy-production pathway regulating genes. X-axis denotes non-stress and stress conditions. Y-axis denotes the gene models of energy-production pathway genes. Refer Table S4 for details of energy-production pathway genes.

Low oxygen conditions give rise to inadequate number of ATPs for energy-utilizing pathways. However, substrate-level ATP production instigates the availability of glucose through sucrose breakdown, thereby maintaining glycolytic flux (Fig. 3). Sucrose is degraded into glucose by the means of invertase and sucrose synthase (SUS), where the former was highly differentially expressed with upregulation upto 48.6-fold change in waterlogging conditions (Supplementary Table S4). *SUS* gene was also up-regulated but with a lower expression in comparison to *invertase* gene (Supplementary Table S4). These results were in agreement with a previous study in *Arabidopsis* where it was observed that the invertase route is more preferential over SUS pathway under hypoxia conditions^23^. SUS catalyzes the conversion of sucrose into UDP-glucose and invertase catalyzes the same into simply glucose where the higher upregulation of *SUS* gene suggest a higher requirement of glucose in the form of UDP-glucose for ATP generation. Glucose generated from sucrose breakdown is utilized by glycolysis which produces ATP used by energy-consuming pathways. Our study observed the expression of nine glycolytic enzymes in waterlogged roots of maize where hexokinase (7) followed by Phosphofructokinase (PFK) (6) noted maximum number of up-regulated encoding genes (Fig. 3, Supplementary Table S4). Amongst all genes, *hexokinase* was the most highly expressed and up-regulated (35.6-fold) ones implicating a strong metabolic role that maintains glycolytic flux in waterlogging conditions. High upregulation of hexokinase in waterlogged roots of maize observed in this study was in concordance with that of a previous study in maize^24^. In addition, the gene sequence of differentially expressed hexokinase mapped four SNPs in its vicinity (Table 2) which suggested that there could possibly be stress-specific markers present in the DEGs in waterlogging sample. PGI, PFK, aldolase, TPI, GAPDH, PHGDH, PGK, and enolase were differentially expressed with upregulation upto 8.8-fold change in waterlogging conditions. PFK uses ATP and PPi to phosphorylate fructose-6-phosphate to fructose-1,6–bishosphate, where PPi-based phosphorylation is studied to be induced in oxygen deficiency stress^25^. The end product of glycolysis is pyruvate, which is fermented into lactate and ethanol through two pathways: one pathway includes the catalyzation of pyruvate into lactate through lactate dehydodrogenase (LDH) and the same into ethanol by the action of pyruvate decarboxylase (PDC) and alcohol dehydrogenase (ADH) (Fig. 3). *ADH* was the highest up-regulated (7.6-fold) DEG amongst the three fermentative genes. The highest number of genes (7) encoding ADH suggested that ethanol fermentation provides more NAD^+^ to maintain glycolysis. In chrysanthemum, the cDNA expression of ADH was accelerated upto 7-fold change under waterlogging stress^26^.

**Table 2:** Polymorphisms identified in the genes expressed in non-stress and stress samples by comparing with B73.

Gene ID	Chr.	Annotation	SNP Position	Non-stress	Stress
AC177897.2_FG002	1	E3 ubiquitin ligases_RING Finger	260370791	—	TATACACCCTTGA (Insertion)
			260370793	—	CACCCTTGAA (Insertion)
			259810372	A (Transition)	—
			259821963	C (Transition)	—
			259824523	TA (Deletion)	—
			260370929	CCAGGGATGGGTG (Insertion)	—
			260371984	A (Transition)	—
GRMZM2G018398	4	ERFVII	66959771	—	CTATTCCAGATCTT (Insertion)
			175831328	CGTG (Insertion)	—
			175845649	—	A (Transversion)
			176049264	—	ATCTTGC (Insertion)
			176049686	—	C (Deletion)
			176207473	—	GGAGAT (Insertion)
			176207558	—	C (Deletion)
			176214503	—	G (Deletion)
			176337735	—	GCT (Insertion)
			176344863	A (Transversion)	—
			176362669	—	TGTG (Insertion)
			176362875	—	T (transversion)
			135901138	—	TGGA (Insertion)
GRMZM2G046686	6	Hexokinase-1	135901645	—	TCAACGACCT (Insertion)
			136160815	C (Transition)	—
			136170494	T (Transversion)	—
			136195092	—	TC (Insertion)
			136195530	—	C (Deletion)
			136400315	—	TG (Deletion)
GRMZM2G094523	3	Expansins	34262921		TA (Deletion)
			34262924	—	GGGACA (Insertion)
			34263003	—	ACAGGCAC (Insertion)
			34263420	CAAAAA	CAAA (Insertion)
			34263423	—	GAAAAAA (Insertion)
			60226516	—	A (Transition)
GRMZM2G467435	3	Polygalacturonases	60323319	—	G (Transversion)
			60327633	—	G (Transition)
			60330822	—	TGG (Insertion)
			60330903	—	TGCA (Insertion)
			60333441	—	C (Transition)

### Programmed Cell Death (PCD) and Aerenchyma Formation

**Figure 4:**
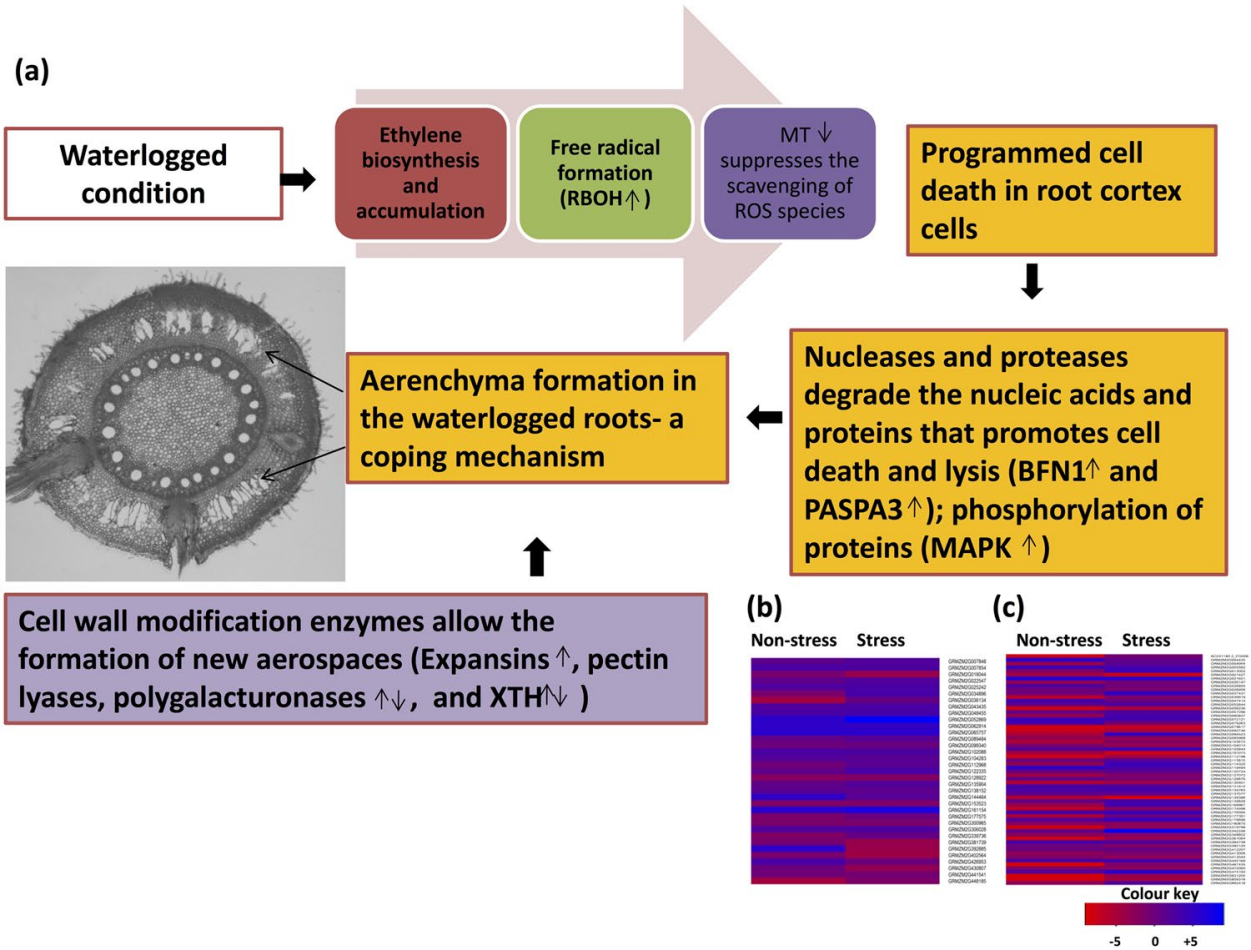
Programmed cell death (PCD) of root cells leading to aerenchyma formation regulated in waterlogging tolerant genotype under stressed conditions. (**a**) PCD pathway and aerenchyma formation during waterlogging stress. Here arrows denote the expression level of waterlogging responsive genes in maize roots. Expression level marked here includes greater than 2 fold change. Refer the paper for abbreviations. (**b**) Heatmap of PCD and (**c**) aerenchyma formation genes. X-axis denotes non-stress and stress conditions. Y-axis denotes the gene models. Refer Table S5 and Table S6 for details of PCD and aerenhcyma formation genes.

In hypoxic conditions, oxygen uptake between roots and shoots is an important aspect for plant survival. PCD progression in hypoxic conditions takes place in root cortical cells which allows the development of gas spaces called aerenchymous tissue^7, 27^. Role of PCD was investigated for their transcript abundance in waterlogged roots of maize. It was evident from the study that ethylene and cell death occur in consequence to waterlogging conditions. Ethylene synthesis gene *ACS* and PCD regulating genes were highly differentially expressed in response to waterlogging conditions (Fig. 4). The transcript abundance of *ACS* and *RBOH* in these conditions is suggestive of the fact that ethylene formation promotes free radical formation by RBOH (Fig. 4). When free radicals are formed, their scavenging occurs by the action of MT but in low oxygen conditions, these free radicals promote the formation of aerenchyma which is preceded by the downregulation of *MT* gene^8^ (Fig. 4). However, in our study, two *MT* genes were down-regulated up to 7-fold in waterlogging conditions (Fig. 4, Supplementary Table S5). Cell wall of root cortex is degraded by the action of nucleases and proteases. This was evident from our study which noticed high expression of *BFN1* and *PASPA3* in waterlogged roots of maize (Fig. 4). It has been studied that these hydrolases are those PCD-regulating genes that are expressed during lateral root cap development^28^. Such hydrolytic enzymes prepare the plant cells for their death and lysis where PCD plays a metabolic role in such conditions (Fig. 4).

In consequence to PCD, cell wall modification takes place in root cortical cells that lead to the development of gaseous spaces (Fig. 4)^29^. These spaces are called aerenchyma which allows an unobstructed oxygen transfer between roots and shoots. Cell wall is made up of pectin which contains homogalacturonan that is hydrolyzed by polygaalcturonases. Depolymerization of pectin is made accessible to these degrading enzymes by reducing the tension in cell wall done through expansins. The transcript abundance of cell wall modification genes was investigated in waterlogged roots of maize where it was found that encoding genes *expansins* (41.8-fold), *polygalacturonases* (16.1-fold), and *XTH* (10.8-fold) were highly differentially expressed and up-regulated by more than 10 folds (Fig. 4, Supplementary Table S6). These highly expressed cell wall modifying aerenhcyma formation genes (GRMZM2G018398, GRMZM2G094523, GRMZM2G467435) mapped 12 SNPs in their vicinity explaining the genetic variability in aerenchyma formation genes in maize.

#### Ethylene-Responsive Pathways

**Figure 5:**
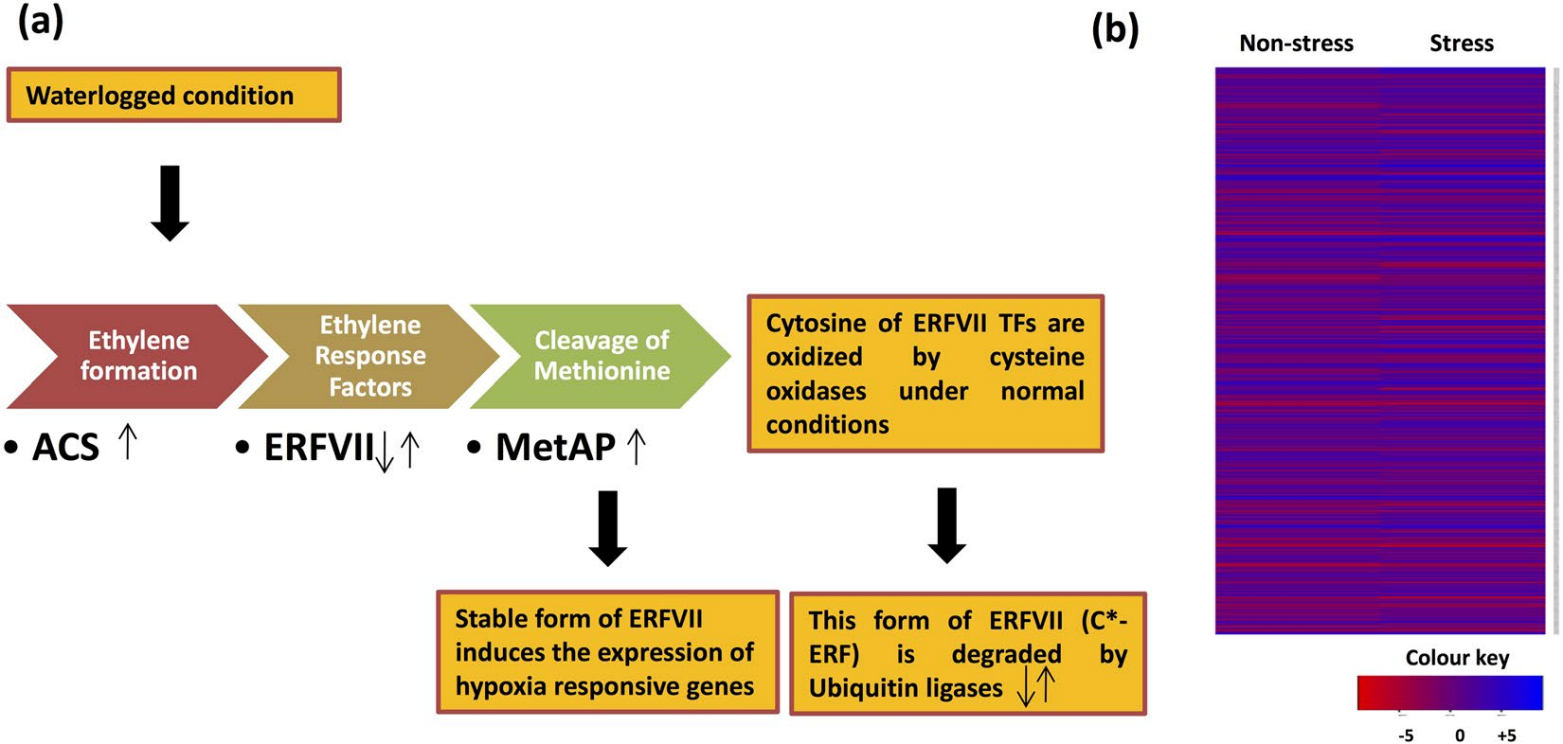
Ethylene-responsive pathways regulated in waterlogging tolerant genotype under stressed conditions. **(a)** Ethylene-responsive pathways regulated during waterlogging stress. Here arrows denote the expression level of waterlogging responsive genes in maize roots. Expression level marked here includes greater than 2 fold change. Refer the paper for abbreviations. **(b)** Heatmap of ethylene-responsive pathway regulating genes. X-axis denotes non-stress and stress conditions. Y-axis denotes the gene models of ethylene-responsive pathway genes. Refer Table S7 for details of ethylene-responsive pathway genes.

Ethylene plays a fundamental role to several tolerance mechanisms in waterlogging conditions. Two ethylene synthesizing *ACS* genes were highly up-regulated upto 2.6-fold (Fig. 5, Supplementary Table S7). It has been studied that group VII of ERF TFs remain stable under low oxygen conditions. In *Arabidopsis*, there are five family members of ERFVII Hypoxia Responsive ERF1 (HRE1), HRE2, Related to AP2.12 (RAP2.12), RAP2.2, and RAP2.3 that are noted to be responsive to hypoxia^21, 22, 30^. In maize, eight ERFs belong to group VII out of which three were up-regulated (highly expressed at 2.8-fold) in waterlogging condition (Fig. 5). Interestingly, genetic variants including 11 SNPs were mapped in a differentially expressed ERFVII gene (GRMZM2G018398) in waterlogging condition. Effect of these SNPs would depend on their mapping in the expressed gene, for instance, SNPs mapped in the intron would affect alternate splicing events^31^ and those mapped in the exon or spliced transcript will be expressed at the post-transcriptional levels in the genome^32^. In addition, ERFVII consists of a methionine-cytosine-initiating sequence which is turned into stable form by the action of MetAPs that cleave methionine from the initiating sequence of TF (Fig. 5). In waterlogging conditions, these TFs remain stable and induce hypoxia-responsive genes. *ADH* is one of the hypoxic genes that is induced by stable ERFVII TFs^33^ and in this study *ADH* was highly differentially expressed in waterlogged roots of maize (Supplementary Table S4).

ERFVII TFs are ubiquitous and are degraded *via* N-end rule pathway under normal oxygen conditions^13^. ERFVII is degraded by a series of events catalyzed by three enzymes: Arginyl-tRNA-protein transferase (ATE), E3 Ubiquitin ligases, and 26 S proteasomes (Fig. 5). In waterlogging conditions, *ATE* was noted for low expression levels. However, varying forms of E3 Ubiquitin ligases and 26 S proteasomes were expressed in waterlogging conditions (Fig. 5) but since these enzymes also cleave non-stress proteins, so for investigating specific hypoxic responsive degradable enzymes mutant lines should be used to be tested upon.

### Crosstalk of Different Pathways under Waterlogging Condition

Maize is a genetically diverse crop for which waterlogging is a major arresting factor in subtropical regions. Species with replacement rooting systems positioned near or at the shoot base are tolerant of waterlogging conditions^34^. In maize, stimulation of outgrowth of root primordia at the shoot base is one of the mechanisms for generating the replacement root system^35^. Ethylene hormone induces the emergence of root primordia in waterlogging conditions^6^. This hormone even more promotes the formation of aerospaces called aerenchyma which improves the diffusion of oxygen from roots to shoots. This strategy is known as low oxygen escape strategy^36, 37^, an expensive strategy which exploits the carbohydrate reserves for a higher fitness of the plant^38^. These carbohydrate reserves come from sucrose metabolism and glycolysis where the same is maintained by energy budget provided by fermentative pathways.

**Figure 6:**
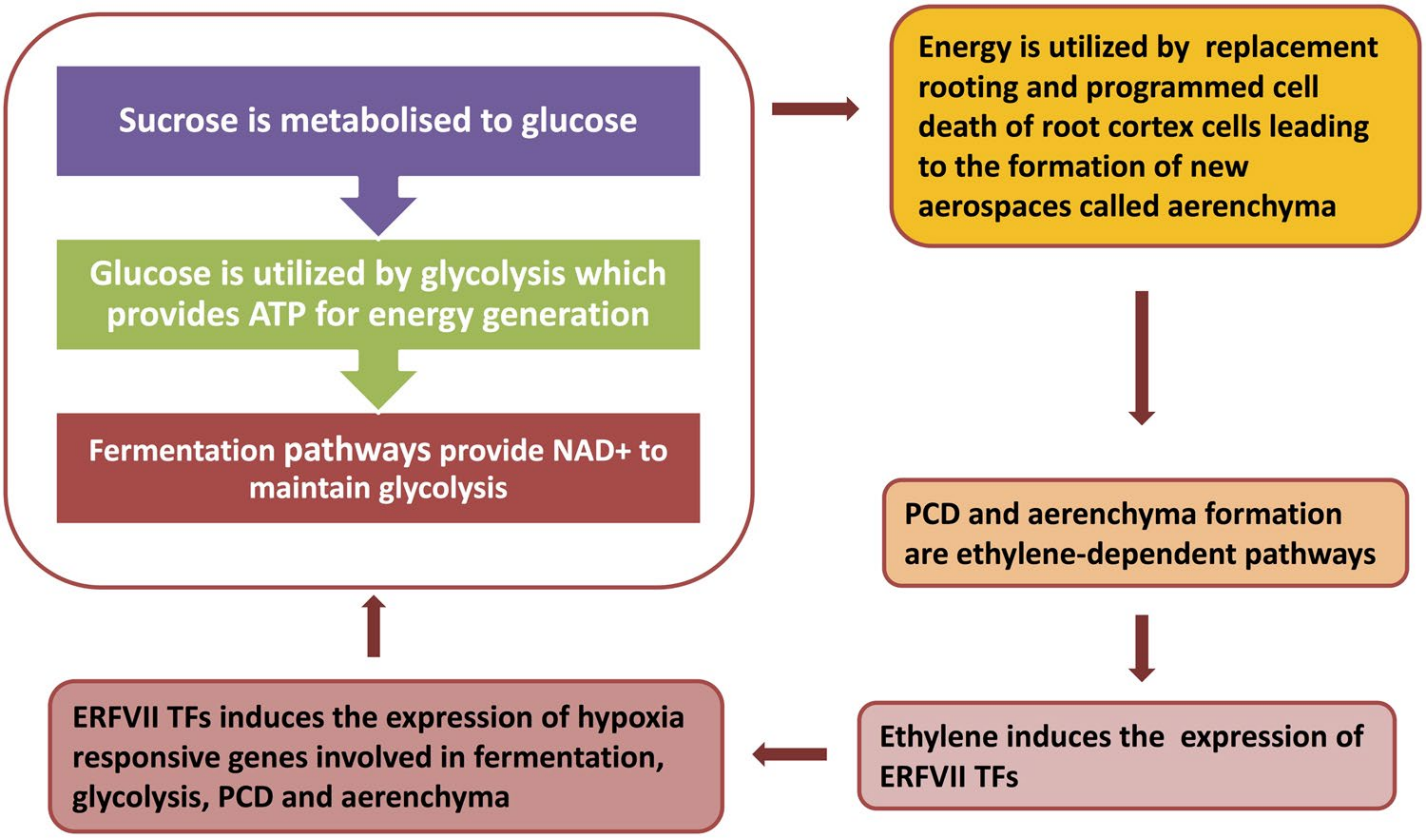
Crosstalk of different pathways regulated in waterlogging tolerant genotype under stressed condition to sustain the biological activities. Water-logging tolerance governed by interactions among several metabolic events *viz*., glycolysis, programmed cell death, aerenchyma formation.

Ethylene stimulates the aerenchyma formation and ethanolic fermentation in waterlogging condition^26^. This hormone stimulates an Ethylene Response Factor (ERF) transcription factor which further activates the hypoxia responsive genes including the ones which encode fermentative enzymes ADH and PDC^21^, cell wall modification genes leading to the formation of aerenchyma *XTH* and *expansins*, PCD regulating genes *RBOH*, sucrose metabolism regulating genes, and glycolytic genes *PFK* under low oxygen conditions^39^. It could be said group VII of ERF transcription factors regulates a subset of waterlogging-responsive pathways. Fermentation is known to provide NAD^+^ to maintain glycolysis and glycolysis further provides ATP to provide energy to the plant for emergence after low-oxygen conditions accumulated in waterlogging. These carbohydrate reserves that create a positive energy budget in a plant promote ethylene-dependent pathways in low oxygen conditions. Maize aerenchyma tissue was noted for the expression of energy production pathways regulating genes–fermentative pathway (ADH and LDH) and glycolysis (PFK), and ethylene-dependent pathways regulating genes–PCD (ACC oxidase and RBOH); and cell wall modification genes leading to the formation of new aerospaces (polygalacturonase, XTH, and expansins)^40^. In addition, the formation of aerenchyma tissue in waterlogged roots of tolerant maize was observed under microscopy (Fig. 4). This indicated that the genes regulating energy production and ethylene responsive pathways were actively involved in aerenchyma formation in roots which is one of the adaptive mechanisms. In addition, these pathways are induced by ethylene and responsive factors through a cross-connected network (Fig. 6). Gibbs *et al*.^41^ had studied the response of mutants of an N-end rule pathway where ERFVII, ACS, MetAP, ATE, and ubiquitin ligases are pathway substrates. They also investigated the hypoxic response of energy production and ethylene regulating genes in these N-end rule mutants. Our study investigated the differential expression of N-end rule pathway substrates in ethylene-responsive pathways and energy production pathways in waterlogging condition. This implicated that the inter-connectivity of energy production, aerenchyma formation, and ethylene responsive pathways regulating genes towards waterlogging tolerance (Fig. 6).

### Importance of Polymorphism in the Expressed Sequences

Polymorphism from the expressed genes sequenced in stress and non-stress samples determine the functional association of candidate genes to complex traits promoting stress tolerance. Many of these SNPs including insertions and deletions were mapped in genes regulating various waterlogging tolerance pathways. For instance, 11 SNPs were mapped in ERFVII gene (GRMZM2G018398) (Table 2) that was highly up-regulated and differentially expressed during waterlogging condition thereby promoting stress tolerance through ethylene-responsive pathways in maize. In addition, SNPs were also mapped in highly differentially expressed and up-regulated genes regulating aerenchyma formation (*XTH* and *polygalacturonases*) and energy production pathways (*hexokinase*) (Table 2). The polymorphisms found in in transcripts of non-stress and stress samples arose from post-transcriptional RNA sequence modification called RNA editing that regulates the alternative splicing of mRNA and amino acid substitutions. RNA editing sites have been detected in humans and estimated to be more than 300 thousand but the same in maize has been done for five genomic regions present upstream of the liguleless-1 gene, male fertility genes, and 49 acetolactate synthase genes^42^. But the total RNA editing sites in the maize genome is still unknown. It has been studied that RNA editing events are specific to conditions^43, 44^ as a result of which different mRNAs are produced in different conditions. RNA editing is a powerful tool to enhance transcript expression for the stress tolerance regulating genes. A deeper study of RNA editing sites in stress tolerance genes can allow the manipulation of genetic sequences which in turn enhances the stress tolerance level in maize.

## Conclusions

Whole genome-RNA sequencing of a maize genotype under waterlogged stress revealed differential expression of genes. The non-stress and stress samples also revealed the variation at RNA-sequence level which could be the reasons for variation in the expression level. Cross-talking of fermentative, sugar metabolism, and glycolytic pathways led to the programmed cell death and aerenchyma formation in root cortical cells. Our experiment uncovered the gene regulatory network rearing tolerance mechanisms in maize *via* adaptive traits such as aerenchyma formation and ethylene formation. Genes and genetic variants identified in various key functional pathways could assist the breeding of waterlogging tolerant maize genotypes.

## Materials and Methods

### Plant Materials and Stress Treatment

An inbred line HKI1105, tolerant to waterlogging stress, was chosen for the experiment. Two sets of disposable plastic cups, with five in each, (250 cm cups perforated at the base at four points with an orifice of ~5.0 mm diameter) were filled with pot mixture up to 220 cm of its volume. Seeds were sown in the cups by applying optimal water and the seedlings were allowed to grow till three leaf stages (27 °C/24 °C day/night and 16 h/8 h light/dark). After the three leaf stage, one of the sets was kept in a plastic tray (60 × 30 × 15 cm^3^) and water was added into the plastic tray above the level of plastic cups to induce the waterlogging stress to the seedlings. The second set was maintained as a non-stress (control) under optimal water condition. Five days after the stress^45^ root tissues of stressed and non-stress plants were isolated and immediately frozen in liquid nitrogen.

### RNA Sequencing Library Construction and Sequencing

Total RNA was extracted from the two biological with two technical replicates of stressed and non-stressed root samples separately and purified using the RNeasy mini kit (Qiagen, Hilden, North Rhine-Westphalia, Germany). Quality and quantity of the isolated RNA was checked using a NanoDrop 1000 spectrophotometer (Thermo Scientific, Wilmington, Delaware, USA) and denaturing agarose gel electrophoresis, respectively. Two technical replications of both samples were maintained and used for quality estimation and reproducibility in Illumina RNASeq. mRNA was extracted and purified from total RNA using oligo(dT) attached magnetic beads. The isolated mRNA was fragmented and primed for cDNA synthesis. Fist-strand and second-strand cDNA were synthesized and end repair was performed that converted the overhangs from fragmentation into blunt ends. Next, 3′ ends of the blunt fragments were adenylated for further adapter ligation. Adapters were ligated to the ends of dscDNA for easier hybridization of sample onto a flow cell. The dscDNA synthesized was amplified using PCR for the availability of an adequate amount of sample for sequencing. cDNA templates were normalized to 10 nM and pooled in equal volumes in 96-well PCR plate. After normalization and pooling of cDNA templates, libraries were then added to the flow cells of Illumina Hiseq for sequencing.

### Identification of Differentially Expressed Genes (DEGs)

To evaluate the gene expression profiling, filtered reads were mapped to the B73 maize genome using CLC Genomics Workbench (https://www.qiagenbioinformatics.com). Mapping of the sequence reads to the reference genome was normalized by exon length to ensure unique matches. Normalized gene expression values were evaluated using CLC Genomics Workbench which utilizes paired sequence reads. Paired reads have an advantage over single reads as more combinations of exons are identified with paired reads that act as unique for each splice variant. Normalized gene expression values are represented in reads per kilobases in million (RPKM), a normalized form of total number of reads mapped to the gene. RPKM values were transformed to log_10_ and plotted in heat maps through R.

### Functional Annotation and Classification

We had assigned putative functions to the RNAs sequenced in non-stress and stress samples by performing BLASTX against maize (*Zea mays*). Protein matches of e-value cutoff of 1e^−10^ were considered for gene ontology (GO) annotation using Blast2GO^46^. GO classification of these protein matches were assigned to the transcripts sequenced in both samples.

### Polymorphism Survey

Single nucleotide polymorphisms (SNPs) were identified using Sequence Alignment/Map (SAMtools) and annotated using SNP effect predictor (snpEff). SAMtools were used to filter SNPs and short INDEL sequence variants^47, 48^. Genetic sequence variants were annotated based on their genomic locations and coding effects were predicted using snpEff^49^. Annotations of sequence variants included intronic, untranslated region, upstream, downstream, splice site, and intergenic regions was conducted. Coding effects included synonymous or non-synonymous amino acids replacement.

### Root Section Analysis

Fresh roots from the stressed plant were collected and a thin section was prepared without mounting in wax to understand the formation of aerenchyma. The cross root sections were observed through a Leica M205FA microscope (Leica Microsystems, Wetzlar, Hesse, Germany). The images of the root sections were captured with an inbuilt camera (DFC425C).

### Validation of RNA-Sequencing Expressed Genes with Quantitative Real Time PCR (qRT-PCR)

DEGs specific to waterlogging-responsive pathways including energy-production, programmed cell death (PCD), aerenchyma formation, and ethylene responsive pathways were validated with qRT-PCR. Primers of these waterlogging-responsive genes were synthesized using IDT software (http://eu.idtdna.com) (Supplementary Table S8).

### Data Deposition

The Illumina RNA-sequencing reads of *Zea mays* under control and waterlogging stresses were submitted to NCBI Sequence Read Archives under the project PRJNA377604.

## Electronic supplementary material

Supplementary information
