# Supplementary material for: RNAseq revealed the important gene pathways controlling adaptive mechanisms under waterlogged stress in maize
Source: Sci Rep. 2017 Sep 8;7:10950. doi: 10.1038/s41598-017-10561-1 (PMC5591269; doi:10.1038/s41598-017-10561-1)
Supplement: Supplementary file 1 — Supplementary information [file 41598_2017_10561_MOESM1_ESM.pdf]

Supplementary Information

RNAseq revealed the important gene pathways controlling adaptive mechanisms under waterlogged stress in maize

Kanika Arora[1, 2], Kusuma Kumari Panda[2], Shikha Mittal[1], Mallana Gowdra Mallikarjuna[1], Atmakuri Ramakrishna Rao[3], Prasanta Kumar Dash[4], Nepolean Thirunavukkarasu[1]*

Table S1. RNA sequencing yield and mapping results of HKI 1105 roots under non-stress and waterlogging stress conditions.

Treatment	Number of paired reads	% of mapped reads	Genes RPKM>0	Genes RPKM>1	Genes RPKM>2	Genes RPKM>5
Non-stress	4,77,17,398	88	21364	12633	9092	4381
Stress	4,49,04,070	84.073	21364	13872	10481	5396

Table S2. Characteristics of single nucleotide polymorphisms (SNPs) mapped in maize roots under non-stress and waterlogging stress conditions.

Genomic regions	SNPs mapped in non-stress sample		SNPs mapped in stress sample	
	Read counts	Rate (%)	Read counts	Rate (%)
Downstream	66,880	13.93%	60,365	11.08%
Exon	47,941	9.99%	40,133	7.37%
Intergenic	6,106	1.27%	4,309	0.79%
Intron	78,945	16.45%	43,845	8.05%
Splice site acceptor	42,667	8.89%	62,319	11.44%
Splice site donor	51,220	10.67%	87,447	16.06%
Splice site region	1,02,022	21.26%	1,66,235	30.52%
Transcript	226	0.05%	215	0.04%
Upstream	48,503	10.11%	49,902	9.16%
UTR 3´	21,827	4.55%	18,751	3.44%
UTR 5´	13,662	2.85%	11,167	2.05%
Total	4,79,999	–	5,44,688	–

Table S3A. Amino acid substitutions in SNPs identified in non-stress expressed transcripts. Rows represent amino acids in B73 maize genome while columns represent changed amino acids. Red colour indicates more changes happened and grey colour represents diagonals.

	*	-	?	A	C	D	E	F	G	H	I	K	L	M	N	P	Q	R	S	T	V	W	Y
*	**34**	5		3	5	2	5	20	6	3	1	3	31				37	48	8	3	5	9	14
-	722		15,994	1,513	669	1,458	1,803	886	1,652	703	2,079	2,443	3,292	699	1,670	957	873	1,474	1,771	1,524	1,881	459	805
?																							
A	3	118		**1,496**	19	39	205	1	188	20	39	46	62	1		140	8	54	695	128	273	41	35
C	53	15			**395**	2		115	1		1	26	10	13	26		4	111	111		25	33	43
D	23	65		104	50	**865**	395		377	25	16	4	26	6	110	13	34	25	3	5	155		277
E	606	206		113	1	510	**1,809**	2	380		3	461	9	160	15	3	79	12	6	21	270	50	3
F	61	91		7	22	1		**208**	18	1	34	5	471	2	6	10		7	150	27	158	3	10
G	80	110		189	209	115	158	1	**1,797**	14	3	24	19	70	15	1		92	61	22	768	123	57
H	1	11		62		13	2	1		**214**		4	47		32	12	133	87	4		1	2	181
I	14	99		31	1	52	3	37	26	2	**723**	59	117	93	116	4	68	96	132	319	367	18	11
K	99	391				1	460		2		63	**1,428**	14	57	405	6	82	345	89	36		2	58
L	77	141		10	160	2	24	417	18	225	238	27	**1,640**	35	16	542	97	31	527	18	95	72	63
M		50		1		5		2	6	9	200	63	34	**283**	71	1	1	12	23	92	49	4	65
N	12	62				204	3		2	45	209	312	1	57	**607**	2	9	6	173	108			19
P		65		121	4					56		10	224		2	**1,044**	70	33	205	158	12		
Q	305	75		6		24	19	5	8	298	9	33	108	3	7	55	**2,238**	169	18	6	5	2	1
R	37	102		9	32	1	40		154	59	29	61	210	144	6	36	21	**1,622**	449	44	9	49	5

**S**	139	259		30	169	3	6	203	258	4	295	48	341	2	46	276	3	161	**2,120**	342	6	1	7
**T**		33		583	7		69		6	7	497	69	11	32	108	40		71	27	**847**	3		1
**V**	21	249		632	33	132	196	130	255	68	248	108	381	113	71	9	63	293	136	33	**1,539**	16	9
**W**	67	7			68	69		2	10	1		3	49					148	1			**46**	
**Y**	71	69		14	98	40	6	39	12	114	70	2	59	1	60		18	9	31	14	10	7	**194**

*=termination codon
–
=gap
?=unknown

Table S3B. Amino acid substitutions in SNPs identified in stress expressed transcripts. Rows represent amino acids in B73 maize genome while columns represent changed amino acids. Red colour indicates more changes happened and grey colour represents diagonals.

	*	-	?	A	C	D	E	F	G	H	I	K	L	M	N	P	Q	R	S	T	V	W	Y
*	105	22	1	8	21	3	1	33	5		1		15					3	53	6	12	30	42
-	1,377		25,101	2,804	1,714	1,979	3,255	1,974	3,289	996	2,896	3,784	5,597	1,328	2,102	1,782	2,308	3,861	3,453	2,899	2,886	765	1,479
?																							
A	36	79		1,229	13	97	112	16	323	56	44	137	69	10	21	119	48	117	140	101	130	42	30
C	33	34		7	332			21	5			13	36	14	25	6		111	23		11	26	29
D	75	73		48	3	944	323	19	67	104	30	25	55	8	81	14	36	64	21	26	79		115
E	162	191		158	18	256	2,925	11	197		32	65	28	21	140	9	124	59	61	21	241	75	82
F	3	34		1	146	9	5	211	27		26	1	203	9	17	4		11	196	74	57	12	60
G	68	134		190	36	115	227	3	1,745	14	7	75	47	44	19	4	20	141	85	8	295	98	12
H	1	7		37		6	5	1		204		1	8			5	85	42	14	1		1	29
I	45	49		58		57	63	4	26	18	549	99	77	82	44	104	92	98	20	159	131	3	174
K	23	128			2	39	201	13	4	27	33	3,021	27	15	499	11	41	123	48	19	14	3	20
L	21	87		7	159	7	27	211	14	44	99	54	2,136	14		137	41	33	272	56	58	18	48
M	22	78			1				3	10	358	60	24	397	5	2	14	127	120	281	14		63
N	16	50		12		39			12	3	158	233	17	37	334	1	1	24	61	22	4	2	65
P	7	34		70	18	6			4	159	3	4	193		105	753	130	15	45	27	8		1
Q	11	111		8	10	18	38	9	10	251	13	23	156			84	3,071	29	34	95	5		3
R	16	12		11	108	8	35	9	121	31	25	135	55	18	13	18	13	1,6	249	38		4	2

		3																10					
**S**	100	133		27	110	78	12	135	39	12	105	127	62	56	55	104	5	175	**2,363**	82	13	15	28
**T**	3	62		265		24	69	3	80	31	160	50	27	73	41	5	2	18	12	**1,075**	3	2	12
**V**	89	160		418	91	222	220	67	249	54	147	289	276	152	171	37	93	157	183	46	**1,698**	22	17
**W**	131	7		6	198			5	3	3	1	2	23	2	1			18	2		13	**120**	4
**Y**	69	46		18	75	28	2	27	11	83	40	4	401	1	17	1	12	65	33	14	35	8	**226**

*=termination codon

–=gap

?=unknown

Table S4. Maize gene expression of Energy-Production Pathway related genes.

Gene name	Gene ID	Annotation	Root (Fold change)
Lactate dehydrogenase (LDH)	GRMZM2G128929	L-lactate dehydrogenase	-3.212497736
	GRMZM2G136072	Glyoxylate reductase	1.173986375
	GRMZM2G074282	hydroxypyruvate reductase	-1.559328686
	GRMZM2G159587	Glyoxylate reductase	-4.088633483
	GRMZM2G049811	Uncharacterized protein	1.290277478
Alcohol dehydrogenase (ADH)	GRMZM2G442658	Alcohol dehydrogenase 1	1.432565675
	GRMZM2G152981	Uncharacterized protein	7.581995337
	GRMZM2G149272	GroES-like zinc-binding dehydrogenase family protein	1.335785779
	GRMZM2G139512	Putative alcohol dehydrogenase superfamily protein	-1.600840623
	GRMZM2G154007	Uncharacterized protein	1.625654786
	GRMZM2G008728	Uncharacterized protein	-1.195732434
	GRMZM2G024303	Uncharacterized protein	2.388562704
Pyruvate decarboxylase (PDC)	AC197705.4_FG001	Pyruvate decarboxylase isozyme 1	3.729852545
	GRMZM2G038821	Pyruvate decarboxylase isozyme 2	-1.721529887
	GRMZM2G087186	Pyruvate decarboxylase isozyme 3	1.140414241
Sucrose synthase (SUS)	GRMZM2G152908	Sucrose synthase 2	7.341513957
	GRMZM2G055331	Putative sucrose-phosphate synthase family protein isoform 1	1.97603525
	GRMZM2G008507	Putative sucrose-phosphate synthase family protein	2.568095195
	GRMZM2G318780	Sucrose synthase	2.054476156
	GRMZM2G140107	unknown	-1.923416289
	GRMZM2G462613	unknown	1.411547519
	GRMZM2G318780	Sucrose synthase	2.054476156
Invertase (INV)	GRMZM2G119689	Beta-fructofuranosidase, insoluble isoenzyme 2	48.59578188
	GRMZM2G084940	unknown	1.759401624

	GRMZM2G040843	alkaline/neutral invertase	-1.135731523
	GRMZM2G136139	Neutral/alkaline invertase	-18.17170437
	GRMZM2G139300	Beta-fructofuranosidase, cell wall isozyme	-1.982367749
	AC213432.2_FG004	Uncharacterized protein	-4.008464199
	GRMZM2G022782	Uncharacterized protein	1.042685267
	GRMZM2G118737	Uncharacterized protein	1.184594685
	GRMZM2G175499	Pectinesterase	3.057256184
	GRMZM2G136106	Pectinesterase	15.40857117
	GRMZM2G007277	Uncharacterized protein	-1.032483203
	GRMZM2G115451	Uncharacterized protein	19.07727859
	GRMZM2G170842	Uncharacterized protein	3.0980196
	GRMZM2G084694	unknown	-1.168180995
	GRMZM2G089836	Invertase	19.81102007
Hexokinase	GRMZM2G104081	Uncharacterized protein	3.06837348
	GRMZM2G046686	Hexokinase-1	35.58646199
	GRMZM2G051806	Hexokinase-1	2.262369576
	GRMZM2G467069	Uncharacterized protein	2.31315112
	GRMZM2G432801	Hexokinase-2	1.486296853
	GRMZM2G058745	Uncharacterized protein	1.514821129
	GRMZM2G171373	Uncharacterized protein	12.84047597
Phosphoglucose isomarase	GRMZM2G065083	unknown	1.265869317
	GRMZM2G140614	Glucose-6-phosphate isomerase	2.082124386
Phosphofructokinase (PFK)	GRMZM5G879882	Uncharacterized protein	7.283063621
	GRMZM2G127717	unknown	2.377322409
	GRMZM2G059151	Uncharacterized protein	2.308584577
	GRMZM2G443985	Uncharacterized protein	1.434131083
	GRMZM2G132069	Uncharacterized protein	-1.362877828
	GRMZM2G004932	Uncharacterized protein	2.729003415
	GRMZM2G139360	Uncharacterized protein	2.292942138

	GRMZM2G324471	Uncharacterized protein	-1.068496217
Fructose-6-bisphosphate aldolase (ALD)	GRMZM2G057823	Fructose-bisphosphate aldolase	1.612832182
	GRMZM2G066024	Fructose-bisphosphate aldolase	1.022441779
	GRMZM2G089365	Fructose-bisphosphate aldolase	-1.460226244
	GRMZM2G069195	Fructose-bisphosphate aldolase	-1.060016088
Triose phosphate isomerase (TPI)	GRMZM2G030784	Triosephosphate isomerase	1.231336284
Glyceraldehyde-3-phosphate dehydrogenase (GAPDH)	GRMZM2G051004	Glyceraldehyde-3-phosphate dehydrogenase	3.293304801
	GRMZM2G104632	Glyceraldehyde-3-phosphate dehydrogenase	2.650101596
	GRMZM2G176307	Uncharacterized protein	2.159525483
Phosphoglycerate kinase (PGK)	GRMZM2G003724	Phosphoglycerate kinase	8.804897811
	GRMZM2G382914	Phosphoglycerate kinase	2.107393457
	GRMZM2G047028	Phosphoglycerate kinase	1.85881176
	GRMZM2G089136	Phosphoglycerate kinase	2.934965937
3-Phosphoglycerate dehydrogenase (PHGDH)	GRMZM2G073814	Uncharacterized protein	1.65091834
	GRMZM2G009323	Uncharacterized protein	2.65981288
	GRMZM2G073814	Uncharacterized protein	1.65091834
Enolase (ENO)	GRMZM2G034848	Enolase	1.729533499
	GRMZM2G481529	unknown	-1.20253926

Table S5. Maize gene expression of Programmed Cell Death related genes.

Gene name	Gene ID	Annotation	Root (Fold change)
Bifunctional nuclease 1 (BFN1)	GRMZM2G112968	Putative bifunctional nuclease	3.249426573
Metallothionein (MT)	GRMZM2G099340	metallothionein-like protein type 2	1.247360523
	GRMZM2G402564	Metallothionein2	-5.45151131
	GRMZM2G430807	metallothionein-like protein type 2	-6.814389138
Mitogen-activated Protein Kinase (MAPK)	GRMZM2G007848	Putative uncharacterized protein	1.790329222
	GRMZM2G007854	hypothetical protein LOC100192548	-1.202887822
	GRMZM2G025242	MAPK activating protein Putative uncharacterized protein	1.392441226
	GRMZM2G048455	Putative uncharacterized protein	1.164203155
	GRMZM2G062914	MPK14-putative uncharacterized protein	-1.186950788
	GRMZM2G089484	product :mitogen activated protein kinase 6	1.802451907
	GRMZM2G102088	Putative uncharacterized protein	1.028642368
	GRMZM2G104283	Putative uncharacterized protein	-1.29671871
	GRMZM2G122335	hypothetical protein LOC100279919	3.271765307
	GRMZM2G135904	MAP kinase activity	2.112286091
	GRMZM2G306028	hypothetical protein LOC100277192	1.199385119
Plant aspartic protease A3 (PASPA3)	GRMZM2G036134	hypothetical protein LOC100191400	18.71040785
	GRMZM2G065757	aspartic proteinase oryzasin-1	-1.147203559
	GRMZM2G128922	Aspartic proteinase nepenthesin-1	1.100612226
	GRMZM2G153523	Hypothetical protein LOC100279644	1.141375642
	GRMZM2G177575	Aspartic-type endopeptidase/ pepsin A	2.134520682
	GRMZM2G339736	transcription factor activity	3.852142792
Respiratory Burst Oxidase Homolog (RBOH)	GRMZM2G022547	fae2 protein	1.951894822
	GRMZM2G034896	hypothetical protein LOC100384248	12.16466145
	GRMZM2G043435	product:respiratory burst oxidase-like protein C	1.274008846

	GRMZM2G138152	respiratory burst oxidase protein B	-1.086739183
	GRMZM2G300965	hypothetical protein LOC100381459	1.004067294
	GRMZM2G441541	oxidoreductase activity, acting on NADH or NADPH, with oxygen as acceptor	-1.214738933
	GRMZM2G441541	oxidoreductase activity, acting on NADH or NADPH, with oxygen as acceptor	6.114512369
	GRMZM2G448185	oxidoreductase activity, acting on NADH or NADPH, with oxygen as acceptor	4.12292834

Table S6. Maize gene expression of aerenchyma formation related genes.

Gene name	Gene ID	Annotation	Root (Fold change)
Expansins	GRMZM2G004955	transcription factor activity	6.9705441
	GRMZM2G013002	product:beta-expansin 8	8.909718023
	GRMZM2G021621	Beta-expansin 1a Putative uncharacterized protein	-1.299651537
	GRMZM2G026147	Expansin-like 3 Putative uncharacterized protein	2.568095195
	GRMZM2G026956	hypothetical protein LOC100216633	-1.362877828
	GRMZM2G056236	hypothetical protein LOC100273516	2.934965937
	GRMZM2G072121	hypothetical protein LOC100191303	-1.344208268
	GRMZM2G094523	Putative uncharacterized protein	41.8232646
	GRMZM2G095968	expansin-like 3 (LOC100285844), mRNA	-1.115081859
	GRMZM2G103672	beta-expansin 4	3.668707421
	GRMZM2G105844	alpha expansin2	2.218694488
	GRMZM2G114322	TSA: Zea mays contig17568, mRNA sequence	15.40857117
	GRMZM2G120724	Alpha-expansin 13 Putative uncharacterized protein	1.696777182
	GRMZM2G127072	Putative uncharacterized protein	2.641469343
	GRMZM2G169967	beta-expansin 3	13.73144778
	GRMZM2G176595	product:beta-expansin 6	1.467482969
	GRMZM2G177391	beta-expansin 1a	7.704285585
	GRMZM2G342246	product:beta-expansin 7	20.06782959
	GRMZM2G361064	alpha-expansin 5	5.13619039
	GRMZM2G445169	alpha-expansin 6	1.11472264
	GRMZM2G474194	Beta-expansin 3	4.151432082
	GRMZM5G859316	Beta-expansin 4	3.668707421
Pectin lyases	GRMZM2G005562	Pectate lyase 4	-1.369187447
	GRMZM2G131912	pectate lyase 8	-1.166173811
	GRMZM2G412207	Hypothetical protein LOC100273705	1.170020205
"	GRMZM2G472060	pectate lyase 12	-1.544594871

Polygalacturonases	AC231180.2_FG006	Uncharacterized protein	11.73986375
	GRMZM2G026855	hypothetical protein LOC100273291	-2.756730151
	GRMZM2G037431	polygalacturonase	8.337971412
	GRMZM2G052844	6-phosphofructokinase complex	1.687605414
	GRMZM2G057296	hypothetical protein LOC100272593	1.85881176
	GRMZM2G079617	polygalacturonase (LOC100285007), mRNA	1.467482969
	GRMZM2G092746	Rho guanyl-nucleotide exchange factor activity	2.201224453
	GRMZM2G113815	polygalacturonase (LOC100282607), mRNA	1.875117126
	GRMZM2G119494	Glycoside hydrolase, family 28 Putative uncharacterized protein	-2.504149504
	GRMZM2G130401	Transcribed locus	1.956643958
	GRMZM2G135763	hypothetical protein LOC100273073	1.226552929
	GRMZM2G137077	polygalacturonase (LOC100282589), mRNA	-1.045929496
	GRMZM2G139828	hypothetical protein LOC100191454	2.568095195
	GRMZM2G174598	Putative uncharacterized protein	5.503061132
	GRMZM2G179696	Putative uncharacterized protein	2.297452844
	GRMZM2G348602	Clone 1560552 mRNA sequence	1.04820212
	GRMZM2G467435	hypothetical protein LOC100194076	16.14231265
	GRMZM5G831200	hypothetical protein LOC100194130	5.869931874
	GRMZM5G882418	polygalacturonase (LOC100285112), mRNA	5.936635645
Xyloglucan transglycosylases	GRMZM2G039919	hypothetical protein LOC100382994 (LOC100382994), mRNA	5.869931874
	GRMZM2G060837	Putative uncharacterized protein	10.76154177
	GRMZM2G112198	hypothetical protein	3.668707421
	GRMZM2G128876	Xyloglucan endotransglycosylase/hydrolase protein 8	-2.101103317
	GRMZM2G180870	Glycosyl hydrolases family 16 protein	-1.070832579
	GRMZM2G319798	hydrolase activity, hydrolyzing O-glycosyl compounds	7.337414843
	GRMZM2G364748	Putative uncharacterized protein Xyloglucan endotransglycosylase/hydrolase protein 8 ;	-1.382347511
	GRMZM2G392125	xyloglucan endotransglucosylase/hydrolase protein 15	-1.930743589

GRMZM2G413006	Xyloglucan endotransglucosylase/hydrolase protein 23	1.834353711
GRMZM2G413044	Xyloglucan endotransglucosylase/hydrolase protein 32	-1.533237556

Table S7. Maize gene expression of ethylene-responsive pathway related genes.

Gene name	Gene ID	Annotation	Root (Fold change)
ACC synthase (ACS)	AC197672.3_FG002	Acetyl-CoA carboxylase Fragment	2.662150513
	GRMZM2G377341	Putative uncharacterized protein	1.243284182
ERF VII	GRMZM2G018398	Ethylene response element binding protein Putative uncharacterized protein	2.827321228
	GRMZM2G052667	vacuolar protein-sorting protein 45	-2.903522328
	GRMZM2G053503	ethylene-responsive factor-like protein 1	-6.673401776
	GRMZM2G085964	Ethylene responsive element binding protein 2	-2.84426677
	GRMZM2G110333	ethylene response element binding protein (LOC100283876), mRNA	1.273505335
	GRMZM2G148333	Ethylene response factor Putative uncharacterized protein	-3.196873916
	GRMZM2G169382	ethylene-responsive factor-like protein 1	-2.141665158
	GRMZM2G171179	Putative uncharacterized protein	1.311840836
Methionine aminopeptidase (MetAP)	GRMZM2G002879	Methionine aminopeptidase (EC 3.4.11.18)	1.135791613
	GRMZM2G068982	Methionine aminopeptidase (EC 3.4.11.18)	3.48527205
	GRMZM2G101438	hypothetical protein LOC100280388	-1.125855597
	GRMZM2G113062	hypothetical protein LOC100274208	1.546225957
	GRMZM2G13	Hypothetical protein LOC100191629	1.540857117

	1473		
	GRMZM5G837364	methionine aminopeptidase 1A	-1.116399923
Arginyl-tRNA--protein transferase (ATE)	GRMZM2G160556	hypothetical protein LOC100383012	1.074671871
E3 ubiquitin ligases_SKP1	GRMZM2G029307	hypothetical protein LOC100273625	1.956643958
	GRMZM2G032562	SKP1-like protein 1B	5.319625761
	GRMZM2G052241	LOC100274693	1.467482969
	GRMZM2G074282	Putative uncharacterized protein	-1.559328686
	GRMZM2G101446	SKP1-like protein 1A	-1.268793559
	GRMZM2G109725	Putative uncharacterized protein	1.634242397
	GRMZM2G142825	Putative uncharacterized protein	1.971031046
E3 ubiquitin ligases_RBX1	GRMZM2G035341	RING-box protein 1a	1.117876732
	GRMZM2G057150	hypothetical protein LOC100383383	-1.135731523
	GRMZM2G361074	Putative uncharacterized protein RING-box protein 1a	1.100612226
E3 ubiquitin ligases_Cullin related	GRMZM2G027750	cullin-RING ubiquitin ligase complex	1.150640055
	GRMZM2G126253	cullin-RING ubiquitin ligase complex	2.405899709
	GRMZM2G166089	cullin-RING ubiquitin ligase complex	1.23483323
	GRMZM2G166694	cullin-RING ubiquitin ligase complex	1.199828842

	GRMZM2G168886	cullin-RING ubiquitin ligase complex	1.735924975
	GRMZM2G380184	Putative uncharacterized protein	1.634967438
E3 ubiquitin ligases_RING Finger	AC177897.2_FG002	hypothetical protein LOC100274874	20.17789082
	AC191534.3_FG003	Transcribed locus, moderately similar to NP_001170535.1 hypothetical protein LOC100384551 [Zea mays]	2.130217212
	AC197705.4_FG006	LOC100274105	1.095136544
	AC210013.4_FG009	RING/U-box superfamily protein	1.173986375
	AC211190.4_FG004	unknown protein	1.100612226
	AC212353.4_FG004	hypothetical protein LOC100304207	1.91901619
	AC213654.3_FG001	hypothetical protein LOC100194242	1.351158099
	AC225564.3_FG003	cysteine-type endopeptidase activity	-1.817170437
	AC231747.1_FG001	hypothetical protein LOC100217064	1.028974744
	AC233939.1_FG003	Nitric oxide synthase interacting protein Putative uncharacterized protein	-1.362877828
	GRMZM2G000014	hypothetical protein LOC100274308	1.008894541
	GRMZM2G000114	Protein binding protein Putative uncharacterized protein	1.409277514
	GRMZM2G001024	hypothetical protein LOC100191719	-1.072130558
	GRMZM2G001114	hypothetical protein LOC100191467	1.867705596
	GRMZM2G00	hypothetical protein LOC100191204	12.96276622

2280		
GRMZM2G003656	Putative uncharacterized protein RING finger protein 13	1.517614374
GRMZM2G003725	hypothetical protein LOC100192054 (LOC100192054), mRNA	1.559200654
GRMZM2G004023	hypothetical protein LOC100193216	1.032357205
GRMZM2G004422	hypothetical protein LOC100274167	-1.606248868
GRMZM2G005365	Ubiquitin ligase protein FANCL	2.843248252
GRMZM2G005840	RING-H2 finger protein	2.201224453
GRMZM2G006399	Transcribed locus, moderately similar to XP_002459697.1 hypothetical protein SORBIDRAFT_02g009010 [Sorghum bicolor]	8.438027069
GRMZM2G006428	Putative uncharacterized protein	3.335188565
GRMZM2G007288	hypothetical protein LOC100276421	1.907727859
GRMZM2G009265	Putative uncharacterized protein Ubiquitin ligase SINAT5	2.171874793
GRMZM2G011473	hypothetical protein LOC100216604	-1.022158371
GRMZM2G013892	Putative uncharacterized protein Ubiquitin-protein ligase/ zinc ion binding protein	-1.228509591
GRMZM2G013971	ubiquitin ligase complex	2.170651891
GRMZM2G015409	Putative uncharacterized protein RING-H2 finger protein ATL5F	-1.734571781
GRMZM2G017636	hypothetical protein LOC100279788	-4.088633483
GRMZM2G018070	RING-H2 finger protein	-1.029111829

GRMZM2G018798	TSA: Zea mays contig21068, mRNA sequence	-1.008529592
GRMZM2G019971	DNA binding protein Putative uncharacterized protein	1.467482969
GRMZM2G020574	Putative uncharacterized protein	1.015949747
GRMZM2G020814	hypothetical protein LOC100384141	-1.479439747
GRMZM2G020996	hypothetical protein LOC100191809	1.239770094
GRMZM2G021233	Clone 701858 mRNA sequence	2.016008156
GRMZM2G021498	Putative uncharacterized protein	4.140398375
GRMZM2G021879	hypothetical protein LOC100192713	1.834353711
GRMZM2G022175	hypothetical protein LOC100192058	-1.522175236
GRMZM2G022711	C3H2C3 RING-finger protein	1.06895333
GRMZM2G024690	hypothetical protein LOC100280299	-1.24389643
GRMZM2G025231	cellulose synthase7	3.384788805
GRMZM2G027120	protein binding protein (LOC100283571), mRNA	-44.29352939
GRMZM2G027131	RING finger protein 5	-1.18942065
GRMZM2G027495	hypothetical protein LOC100280311	-2.973551624
GRMZM2G027723	cellulose synthase-2	2.240532032
GRMZM2G02	hypothetical protein LOC100304258	2.034465025

7856		
GRMZM2G028183	hypothetical protein LOC100217233	-1.274235367
GRMZM2G028353	hypothetical protein LOC100280232	4.971364404
GRMZM2G029001	hypothetical protein LOC100276679	1.680779912
GRMZM2G030768	hypothetical protein LOC100383801	5.018791752
GRMZM2G030955	RING zinc finger protein-like	1.773208587
GRMZM2G032071	hypothetical protein LOC100191907	1.485057615
GRMZM2G032821	hypothetical protein LOC100278355	1.467482969
GRMZM2G035601	zinc finger, C3HC4 type family protein (LOC100281133), mRNA	1.504791858
GRMZM2G035785	Protein binding protein Putative uncharacterized protein	-1.802515836
GRMZM2G035821	hypothetical protein LOC100279034	-3.407194569
GRMZM2G035899	hypothetical protein LOC100276255	3.06147309
GRMZM2G036697	hypothetical protein LOC100381407	-1.170923204
GRMZM2G037627	RING-H2 finger protein ATL5F	10.63925152
GRMZM2G039725	S-ribonuclease binding protein SBP1	-3.407194569
GRMZM2G040207	Zinc finger, C3HC4 type family protein	1.704420323
GRMZM2G040803	ring domain containing protein	-1.053132867

GRMZM2G041549	Putative uncharacterized protein Zinc finger, C3HC4 type family protein	-2.271463046
GRMZM2G041561	BRCA1-associated protein	1.54338726
GRMZM2G045084	Putative RING zinc finger domain superfamily protein	-2.95290196
GRMZM2G045286	hypothetical protein LOC100194366	-1.953458219
GRMZM2G046658	hypothetical protein LOC100193782	1.144813521
GRMZM2G047167	zinc ribbon 1	1.56318838
GRMZM2G049070	hypothetical protein LOC100193225	-1.362877828
GRMZM2G049346	hypothetical protein LOC100191632	1.388159565
GRMZM2G049672	Putative uncharacterized protein	2.132003558
GRMZM2G050216	zinc finger, C3HC4 type family protein	-1.362877828
GRMZM2G050774	hypothetical protein LOC100274803	-1.409873615
GRMZM2G051101	Putative uncharacterized protein	1.695195843
GRMZM2G052034	Hypothetical protein LOC100272854	1.109560293
GRMZM2G052344	RING-H2 finger protein ATL2B	1.326378837
GRMZM2G052688	ATBRCA1	2.171478176
GRMZM2G053027	hypothetical protein LOC100194362	-1.640501089
GRMZM2G05	Putative RING zinc finger domain superfamily protein isoform 1	1.271818573

3210		
GRMZM2G053303	LOC100285270	2.934965937
GRMZM2G053511	hypothetical protein LOC100194163	-1.238979843
GRMZM2G053707	unknown	7.337414843
GRMZM2G053909	Putative uncharacterized proteinZinc finger, C3HC4 type family protein ;	1.516399067
GRMZM2G055037	ubiquitin-protein ligase/ zinc ion binding protein	1.467482969
GRMZM2G055643	Putative uncharacterized protein	-1.00014807
GRMZM2G055973	hypothetical protein LOC100274339	2.088341148
GRMZM2G056270	RING finger and CHY zinc finger domain-containing protein 1 (LOC100282790), mRNA	1.931925717
GRMZM2G058450	hypothetical protein LOC100192826	-2.827971492
GRMZM2G059110	Putative RING zinc finger domain superfamily protein	-1.362877828
GRMZM2G061624	Putative uncharacterized protein SINA4 Ubiquitin ligase SINAT4	1.104040925
GRMZM2G061663	Putative RING zinc finger domain superfamily protein	-2.044316741
GRMZM2G061980	Putative uncharacterized protein Zinc finger, C3HC4 type family protein	1.209314669
GRMZM2G062724	Putative uncharacterized protein	-13.35620271
GRMZM2G064580	hypothetical protein LOC100382007	1.021715293
GRMZM2G065893	Hypothetical protein LOC100279516	1.59696676

GRMZM2G066169	hypothetical protein LOC100191553	1.415072863
GRMZM2G066171	hypothetical protein LOC100381983	1.971930239
GRMZM2G068128	Putative uncharacterized protein RING-H2 finger protein ATL2M	-2.877186525
GRMZM2G068239	Putative RING zinc finger domain superfamily protein	-11.58446153
GRMZM2G068590	inhibitor of apoptosis-like protein	-1.168180995
GRMZM2G069162	Putative uncharacterized protein	1.108099384
GRMZM2G069215	Zinc finger, C3HC4 type family protein	1.287054735
GRMZM2G069923	hypothetical protein LOC100383370	-3.167770086
GRMZM2G069928	unknown	1.619291551
GRMZM2G071277	RING zinc finger protein-like	7.337414843
GRMZM2G071602	Putative uncharacterized protein	-1.544594871
GRMZM2G071613	PHD-finger family protein	1.724292488
GRMZM2G072462	hypothetical protein LOC100191292	-1.40881753
GRMZM2G073228	hypothetical protein LOC100193125	-2.516082143
GRMZM2G074254	hypothetical protein LOC100381693	-1.035787149
GRMZM2G075019	Putative uncharacterized protein	1.254461247
GRMZM2G07	Putative uncharacterized protein RING finger and CHY zinc finger domain-containing	1.626498151

7307	protein 1	
GRMZM2G077809	hypothetical protein LOC100279782	1.0723914
GRMZM2G078526	Putative uncharacterized protein Ring domain containing protein	-4.542926092
GRMZM2G080079	Putative uncharacterized protein RING-H2 finger protein ATL1Q	1.677123393
GRMZM2G081060	Zinc finger, C3HC4 type family protein	1.907727859
GRMZM2G081075	Putative uncharacterized protein	1.769611815
GRMZM2G081350	hypothetical protein LOC100382524	1.356919183
GRMZM2G081829	Putative uncharacterized protein	-1.26233766
GRMZM2G081912	hypothetical protein LOC100274494	2.230574112
GRMZM2G081965	hypothetical protein LOC100276574	-1.348063938
GRMZM2G082580	hypothetical protein LOC100191657	2.934965937
GRMZM2G083382	unknown	-1.362877828
GRMZM2G084819	hypothetical protein LOC100193494	1.39488118
GRMZM2G085948	protein binding protein	1.462687273
GRMZM2G087787	Uncharacterized protein	1.399543942
GRMZM2G089466	hypothetical protein LOC100383634	-1.067282445
GRMZM2G092244	hypothetical protein LOC100193667	4.236765345

GRMZM2G095025	Putative RING zinc finger domain superfamily protein	-4.088633483
GRMZM2G095873	protein binding protein	-4.088633483
GRMZM2G096281	Putative uncharacterized protein	-1.362877828
GRMZM2G098637	hypothetical protein LOC100191926	1.577544191
GRMZM2G099238	hypothetical protein LOC100192691	-8.177266965
GRMZM2G101852	Ubiquitin ligase SINAT3	-1.143058823
GRMZM2G102243	unknown	1.238728271
GRMZM2G103245	Putative uncharacterized protein Ubiquitin-protein ligase/ zinc ion binding protein	1.619291551
GRMZM2G105460	RING-H2 finger protein ATL3B	-4.088633483
GRMZM2G105770	unknown	3.660554738
GRMZM2G106377	hypothetical protein LOC100278311	-1.362877828
GRMZM2G107463	Putative uncharacterized protein	-1.54350019
GRMZM2G108084	Zinc finger, C3HC4 type family protein	1.173986375
GRMZM2G108085	RING-H2 finger protein ATL1G	1.399227947
GRMZM2G110423	hypothetical protein LOC100279995	-4.429352939
GRMZM2G111614	Putative uncharacterized protein	-1.492675716
GRMZM2G11	Putative uncharacterized protein	1.385956137

3002		
GRMZM2G113039	Putative uncharacterized protein Ubiquitin-protein ligase/ zinc ion binding protein	-1.265529411
GRMZM2G113137	cellulose synthase 6	2.171239825
GRMZM2G113295	RING zinc finger protein-like	-1.316678579
GRMZM2G116574	hypothetical protein LOC100382051	-1.007344481
GRMZM2G116714	hypothetical protein LOC100193229	2.875473384
GRMZM2G116952	hypothetical protein LOC100279906	2.534743309
GRMZM2G117612	LOC100279085	-1.240994445
GRMZM2G118265	Putative uncharacterized protein	1.822768319
GRMZM2G118344	hypothetical protein LOC100381884	-1.111424354
GRMZM2G118385	Putative uncharacterized protein Ubiquitin-protein ligase/ zinc ion binding protein	1.244170343
GRMZM2G119930	Putative uncharacterized protein Zinc finger, RING-type	-1.282403137
GRMZM2G120816	RING-finger protein like	1.178588238
GRMZM2G122284	RING-H2 finger protein ATL2C	-24.5318009
GRMZM2G122848	hypothetical protein LOC100217052	-12.26590045
GRMZM2G123212	ubiquitin-protein ligase CIP8	-2.044316741
GRMZM2G123660	HEAT/U-box domain-containing protein	-1.141815799

GRMZM2G123791	RNA-binding region-containing protein 1	-1.26375944
GRMZM2G124441	RING finger protein 126	1.926425519
GRMZM2G124701	C-terminal zinc-finger	-1.444001508
GRMZM2G124915	Putative uncharacterized protein Ubiquitin ligase SINAT3	-1.07595618
GRMZM2G125867	ubiquitin-protein ligase/ zinc ion binding protein	1.467482969
GRMZM2G126795	Putative uncharacterized protein	-1.187022624
GRMZM2G129150	RING finger protein 5	2.81267569
GRMZM2G129585	Putative uncharacterized protein	2.527331779
GRMZM2G130167	unknown	7.337414843
GRMZM2G131245	ubiquitin-protein ligase/ zinc ion binding protein	1.948210148
GRMZM2G131591	RNA-binding protein	1.163504354
GRMZM2G133396	Protein binding protein Putative uncharacterized protein	-1.542368629
GRMZM2G134023	hypothetical protein LOC100217252	1.712063463
GRMZM2G135866	Putative uncharacterized protein	2.03817079
GRMZM2G135909	hypothetical protein LOC100304260	12.54697938
GRMZM2G136293	Putative RING zinc finger domain superfamily protein	-1.014783491
GRMZM2G13	protein binding protein	-5.062117645

8997		
GRMZM2G139369	Putative uncharacterized protein	1.391904447
GRMZM2G139941	Protein binding protein	1.222902474
GRMZM2G140160	protein binding protein	1.08130324
GRMZM2G140651	RING-H2 finger protein ATL1Q	-1.168180995
GRMZM2G140924	zinc finger, C3HC4 type family protein	1.467482969
GRMZM2G141216	Zinc finger, C3HC4 type family protein	-1.422133385
GRMZM2G141379	hypothetical protein LOC100383409	-1.163432292
GRMZM2G141596	CONSTANS interacting protein 4	-1.362877828
GRMZM2G141922	Putative uncharacterized protein	-1.417392941
GRMZM2G143644	unknown	1.027238078
GRMZM2G144782	Putative uncharacterized protein RING finger and CHY zinc finger domain-containing protein 1	-7.229178042
GRMZM2G145123	hypothetical protein LOC100194220	18.34353711
GRMZM2G145374	hypothetical protein LOC100381751	-4.088633483
GRMZM2G146267	hypothetical protein LOC100273232	1.427764769
GRMZM2G146354	SINA2 Ubiquitin ligase SINAT2	-2.299856334
GRMZM2G146847	RING-H2 finger protein ATL1R	-1.362877828

GRMZM2G147809	LOC100285109	-2.385036198
GRMZM2G148249	hypothetical protein LOC100276243	1.136782581
GRMZM2G148908	Putative uncharacterized protein Ubiquitin-protein ligase/ zinc ion binding protein	2.678156418
GRMZM2G149028	Putative uncharacterized protein RING finger protein 5	-1.552166415
GRMZM2G151576	CONSTANS interacting protein 4	1.031391332
GRMZM2G151616	hypothetical protein LOC100193862	-1.417392941
GRMZM2G152408	Ubiquitin-protein ligase/ zinc ion binding protein	3.081714234
GRMZM2G152461	RING finger protein 5	-1.703597284
GRMZM2G153250	Putative uncharacterized protein	1.410159415
GRMZM2G153611	hypothetical protein LOC100383096	1.584881606
GRMZM2G154484	Zinc finger, C3HC4 type family protein	-1.362877828
GRMZM2G156415	Putative uncharacterized protein	1.037720099
GRMZM2G157246	Hypothetical protein LOC100191157	-1.362877828
GRMZM2G157855	Putative uncharacterized protein	9.171768553
GRMZM2G157925	Putative uncharacterized protein	-1.039763935
GRMZM2G160966	hypothetical protein LOC100192050	-2.725755655
GRMZM2G16	RUBISCO activase1	1.359016836

2184		
GRMZM2G162356	anaphase-promoting complex subunit 11	-1.58093828
GRMZM2G162949	RING zinc finger protein-like	-1.309431638
GRMZM2G164358	Putative uncharacterized protein RHC1A	1.354599663
GRMZM2G164426	hypothetical protein LOC100280415	-4.391495222
GRMZM2G165044	unknown	-1.817170437
GRMZM2G165063	inhibitor of apoptosis-like protein	-1.192518099
GRMZM2G168200	hypothetical protein LOC100382173	-1.443047112
GRMZM2G168690	Zinc finger, C3HC4 type family protein	-2.521323981
GRMZM2G168707	Putative uncharacterized protein Zinc finger, C3HC4 type family protein	-1.259368119
GRMZM2G169449	hypothetical protein LOC100192025	-1.453736349
GRMZM2G169645	hypothetical protein LOC100279610	1.039467103
GRMZM2G169994	hypothetical protein LOC100273064	4.769319648
GRMZM2G170137	hypothetical protein LOC100193852	1.903761689
GRMZM2G170434	hypothetical protein LOC100273142	-1.061982723
GRMZM2G171232	Putative uncharacterized protein	1.354599663
GRMZM2G171277	Putative uncharacterized protein	-1.362877828

GRMZM2G171561	unknown	1.467482969
GRMZM2G171967	Putative uncharacterized protein RNA-binding protein	1.499384772
GRMZM2G174926	Putative uncharacterized protein	-1.327706787
GRMZM2G175661	hypothetical protein LOC541975	-1.342536367
GRMZM2G176028	RHC1A	1.228590392
GRMZM2G177631	cellulose synthase8	2.538278845
GRMZM2G178522	Putative uncharacterized protein	3.668707421
GRMZM2G180195	Putative uncharacterized protein	1.03343871
GRMZM2G181519	LOC100283384	1.444906307
GRMZM2G300589	hypothetical protein LOC100272564	1.400779197
GRMZM2G305264	hypothetical protein LOC100383341	-1.362877828
GRMZM2G306079	hypothetical protein LOC100191396	-1.226590045
GRMZM2G307823	Uncharacterized protein	-1.748038953
GRMZM2G309063	Ubiquitin ligase SINAT3	1.65091834
GRMZM2G309568	zinc ion binding	3.144606361
GRMZM2G310283	hypothetical protein LOC100280438	1.847941516
GRMZM2G31	ring finger protein	-1.362877828

2078		
GRMZM2G312712	hypothetical protein LOC100279071	1.111729522
GRMZM2G315072	Putative uncharacterized protein	7.484163139
GRMZM2G318220	hypothetical protein LOC100276223	-1.362877828
GRMZM2G318408	RING zinc finger protein-like	-2.385036198
GRMZM2G320399	hypothetical protein LOC100382497	1.030232941
GRMZM2G324111	RING-H2 finger protein ATL5H	3.668707421
GRMZM2G324781	Putative SNF2-domain/RING finger domain/helicase domain protein	-1.196673214
GRMZM2G330684	unknown	-1.362877828
GRMZM2G336761	Putative uncharacterized protein Zinc finger, C3HC4 type family protein	1.271818573
GRMZM2G339151	Putative RING finger U-box domain family protein	1.827431621
GRMZM2G340444	Putative RING zinc finger domain superfamily protein	-34.07194569
GRMZM2G341271	hypothetical protein LOC100274688	1.156529328
GRMZM2G342039	unknown	1.313011077
GRMZM2G343972	hypothetical protein LOC100217033	-2.862043438
GRMZM2G344212	hypothetical protein LOC100193154	-1.121232823
GRMZM2G345544	copine-1	1.691336303

GRMZM2G352891	unknown	-1.898294117
GRMZM2G353091	unknown	4.769319648
GRMZM2G355846	unknown	-1.362877828
GRMZM2G356895	RING-H2 finger protein ATL2M	1.467482969
GRMZM2G359505	Putative uncharacterized protein	1.579139281
GRMZM2G361074	Putative uncharacterized protein RING-box protein 1a	1.100612226
GRMZM2G363253	hypothetical protein LOC100193628	2.934965937
GRMZM2G364612	Putative RING zinc finger domain superfamily protein	-2.149153497
GRMZM2G365888	RING/FYVE/PHD-type zinc finger family protein	1.970231763
GRMZM2G375153	hypothetical protein LOC100191688	-2.725755655
GRMZM2G381386	RNA polymerase T phage-like 1	1.584214568
GRMZM2G383833	hypothetical protein LOC100383546	-1.567309502
GRMZM2G386113	unknown	-2.044316741
GRMZM2G391413	Uncharacterized protein	1.033160655
GRMZM2G392320	hypothetical protein LOC100273800	1.305811116
GRMZM2G393349	protein binding protein	-1.52807514
GRMZM2G39	hypothetical protein LOC100279601	-1.265529411

9901		
GRMZM2G400238	Putative uncharacterized protein	1.989521992
GRMZM2G400268	RING finger protein 5	-1.453736349
GRMZM2G401436	protein binding protein	2.494721046
GRMZM2G402341	hypothetical protein LOC100275703	1.136968786
GRMZM2G409627	hypothetical protein LOC100192498	-1.413354784
GRMZM2G415012	hypothetical protein LOC100191882	1.079344357
GRMZM2G416216	hypothetical protein LOC100384078	-4.088633483
GRMZM2G417089	unknown	2.372009109
GRMZM2G417125	Protein binding protein Putative uncharacterized protein	-1.090302262
GRMZM2G423956	Putative uncharacterized protein	2.288904253
GRMZM2G424582	Uncharacterized protein	1.338398819
GRMZM2G426613	Hypothetical protein LOC100384773	-1.362877828
GRMZM2G428179	RING-H2 finger protein ATL2K	-5.45151131
GRMZM2G433333	hypothetical protein LOC100274776	-1.06143133
GRMZM2G440259	Hypothetical protein LOC100384204	-1.147686592
GRMZM2G445905	cellulose synthase10	2.383192341

	GRMZM2G449355	hypothetical protein LOC100216734	1.473400239
	GRMZM2G449875	RING-H2 finger protein ATL2A	-2.45318009
	GRMZM2G456241	hypothetical protein LOC100383358	4.383635021
	GRMZM2G461447	hypothetical protein LOC100384551	1.781943605
	GRMZM2G464976	hypothetical protein LOC100384168	1.26366589
	GRMZM2G467187	hypothetical protein LOC100192844	-5.45151131
	GRMZM2G468260	Putative uncharacterized protein	-2.611654256
	GRMZM2G477205	Chaperone protein dnaJ 11	-4.936407879
	GRMZM2G478553	Ring-H2 zinc finger protein	1.027238078
	GRMZM2G480106	hypothetical protein LOC100277663	-4.088633483
	GRMZM2G482046	RING-H2 finger protein ATL2M	1.956643958
	GRMZM2G567897	Putative RING zinc finger domain superfamily protein	1.375765283
	GRMZM2G704032	Putative zinc-finger protein Fragment	-1.382773854
E3 ubiquitin ligases_HECT	GRMZM2G021299	hypothetical protein LOC100304346	1.116645138
	GRMZM2G034622	Putative uncharacterized protein	-1.120173557
	GRMZM2G049141	hypothetical protein LOC100382342	1.403226318
	GRMZM2G08	Putative uncharacterized protein	1.441737653

	0439		
	GRMZM2G181378	hypothetical protein LOC100279774	1.279344126
	GRMZM2G331368	hypothetical protein LOC100384278	1.435845691
	GRMZM2G374574	Putative hect E3 ubiquitin ligase Fragment	-1.039025671
	GRMZM2G411536	unknown	1.21790785
	GRMZM2G461948	ubiquitin-protein ligase 7	1.778525554
E3 ubiquitin ligases_BTB	GRMZM2G000261	voltage-gated potassium channel complex	-3.180048264
	GRMZM2G000623	Elongin C	3.668707421
	GRMZM2G000909	hypothetical protein LOC100191308	1.467482969
	GRMZM2G000936	hypothetical protein LOC100192477	3.104674852
	GRMZM2G006424	Hypothetical protein LOC100276863	-1.946968325
	GRMZM2G009724	Putative uncharacterized protein Speckle-type POZ protein	1.645359692
	GRMZM2G017404	Putative uncharacterized protein	2.043994135
	GRMZM2G017486	Putative uncharacterized protein	2.445804948
	GRMZM2G020156	Protein binding protein Putative uncharacterized protein	-13.62877828
	GRMZM2G023346	hypothetical protein LOC100279937	2.384659824
	GRMZM2G027688	hypothetical protein LOC100275864	1.467482969

GRMZM2G029307	Putative uncharacterized protein	1.956643958
GRMZM2G033267	hypothetical protein LOC100191409	-12.26590045
GRMZM2G038691	GAMYB-binding protein	1.400779197
GRMZM2G039280	Ethylene-overproduction protein 1	2.614787835
GRMZM2G040115	Putative uncharacterized protein	7.704285585
GRMZM2G045257	voltage-gated potassium channel complex	1.366277247
GRMZM2G048281	Putative uncharacterized protein	2.132436189
GRMZM2G055785	cellulose synthase catalytic subunit 11	-1.698929895
GRMZM2G058531	hypothetical protein LOC100273151	2.078934205
GRMZM2G059282	Elongin C	-1.424268721
GRMZM2G059865	hypothetical protein LOC100382519	3.250983309
GRMZM2G060765	Hypothetical protein LOC100381943	-1.639712386
GRMZM2G067053	Hypothetical protein LOC100279255	-6.814389138
GRMZM2G074323	LOC100284563	-1.014909021
GRMZM2G076450	Regulatory protein NPR1	1.169627515
GRMZM2G077197	Regulatory protein NPR1	2.934965937
GRMZM2G08	unknown	-1.934407239

5189		
GRMZM2G088086	Putative uncharacterized protein Speckle-type POZ protein	5.869931874
GRMZM2G095510	LOC100282480	1.712063463
GRMZM2G098227	hypothetical protein LOC100279213	-1.081003309
GRMZM2G100946	Uncharacterized protein	1.058816825
GRMZM2G110531	Putative uncharacterized protein	1.035870331
GRMZM2G115162	hypothetical protein LOC100384298	1.981102008
GRMZM2G118082	hypothetical protein LOC100279911	-1.154804877
GRMZM2G128485	hypothetical protein LOC100194212	2.868262166
GRMZM2G130505	BTB/POZ domain containing protein	2.201224453
GRMZM2G142825	Putative uncharacterized protein	1.971031046
GRMZM2G143782	LOC100283633	1.712063463
GRMZM2G148213	hypothetical protein LOC100191618	-1.166173811
GRMZM2G154149	hypothetical protein LOC100280048	-1.409873615
GRMZM2G154437	hypothetical protein LOC100193156	1.801001825
GRMZM2G157379	voltage-gated potassium channel complex	1.124029508
GRMZM2G159161	hypothetical protein LOC100191465	-1.362877828

GRMZM2G162640	hypothetical protein LOC100383502	-1.61841742
GRMZM2G163671	hypothetical protein LOC100217158	1.315674386
GRMZM2G164669	Hypothetical protein LOC100274902	1.510644232
GRMZM2G169782	hypothetical protein LOC100191918	-19.08028959
GRMZM2G172210	hypothetical protein LOC100272403	-1.101395454
GRMZM2G172376	hypothetical protein LOC100274431	-1.530903861
GRMZM2G172506	hypothetical protein LOC100280174	5.13619039
GRMZM2G301803	hypothetical protein LOC100192849	-1.47645098
GRMZM2G320703	Putative uncharacterized protein TD and POZ domain-containing protein 1	1.138564372
GRMZM2G350711	hypothetical protein LOC100279559	1.32234729
GRMZM2G353024	unknown	3.668707421
GRMZM2G397948	unknown	1.362662757
GRMZM2G416184	unknown	-2.180604524
GRMZM2G416964	seven-transmembrane-domain protein 1	-9.540144793
GRMZM2G418031	unknown	2.017789082
GRMZM2G436511	hypothetical protein LOC100278588	1.862574537
GRMZM2G47	Putative uncharacterized protein	-1.362877828

	6637		
E3 ubiquitin ligases_F Box	AC149829.2_FG006	Uncharacterized protein	1.859367625
	AC190750.2_FG014	F-box domain containing protein	-1.06351738
	AC194341.4_FG004	hypothetical protein LOC100279285	-1.233079939
	AC204868.3_FG004	Putative F-box containing family protein	-1.362877828
	AC207342.3_FG007	F-box domain containing protein	1.809895661
	AC214635.3_FG002	LOC100281539	1.086501813
	AC233942.1_FG001	Hypothetical protein LOC100382855	-1.779312719
	GRMZM2G001272	Putative uncharacterized protein	1.427460706
	GRMZM2G001639	hypothetical protein LOC100193825	-2.131680705
	GRMZM2G002427	Ubiquitin-protein ligase	-1.111821386
	GRMZM2G003663	Kelch motif family protein Putative uncharacterized protein	-1.250860472
	GRMZM2G004592	F-box domain containing protein Putative uncharacterized protein	1.281464001
	GRMZM2G005486	F-box domain containing protein Putative uncharacterized protein	1.801001825
	GRMZM2G005622	Putative uncharacterized protein	2.379101176
	GRMZM2G009021	unknown	2.503353299
	GRMZM2G009717	Uncharacterized protein	1.188661205

GRMZM2G010551	Putative uncharacterized protein	1.007096155
GRMZM2G011055	unknown	-64.05525789
GRMZM2G011627	Putative uncharacterized protein	-1.566772936
GRMZM2G012393	F-box protein interaction domain containing protein	2.971653011
GRMZM2G012821	Uncharacterized protein	2.568095195
GRMZM2G016878	hypothetical protein LOC100193490	-1.749026545
GRMZM2G017933	Putative uncharacterized protein	1.99314851
GRMZM2G020366	Putative uncharacterized protein	1.10780577
GRMZM2G020594	F-box domain containing protein	1.808148658
GRMZM2G024806	Putative uncharacterized protein	-1.941068421
GRMZM2G025783	hypothetical protein LOC100384515	-6.294017603
GRMZM2G026229	hypothetical protein LOC100216784	4.402448906
GRMZM2G028014	hypothetical protein LOC100273165	-1.908028959
GRMZM2G028796	Uncharacterized protein	1.100612226
GRMZM2G029039	Kelch motif family protein Putative uncharacterized protein	3.815455718
GRMZM2G029478	F-box domain containing protein	1.058184998
GRMZM2G03	hypothetical protein LOC100192018	-1.362877828

0823		
GRMZM2G033027	hypothetical protein LOC100383592	1.192329912
GRMZM2G034748	unknown	1.033160655
GRMZM2G035243	kelch motif family protein	-1.21019027
GRMZM2G037882	hypothetical protein LOC100277619	3.616297315
GRMZM2G038839	hypothetical protein LOC100383249	-3.407194569
GRMZM2G040182	Hypothetical protein LOC100384416	1.438133309
GRMZM2G041048	LOC100285522	1.614231265
GRMZM2G041959	hypothetical protein LOC100382458	-1.221890466
GRMZM2G042752	unknown	-1.060016088
GRMZM2G044388	hypothetical protein LOC100274439	-3.35359825
GRMZM2G045820	hypothetical protein LOC100279556	1.002183491
GRMZM2G046816	hypothetical protein LOC100193930	4.668726057
GRMZM2G047777	F-box domain containing protein Putative uncharacterized protein	1.222902474
GRMZM2G048661	Putative uncharacterized protein	1.31025265
GRMZM2G048665	RNI-like superfamily protein	-1.238979843
GRMZM2G049364	hypothetical protein LOC100383281	1.260530242

GRMZM2G051090	hypothetical protein LOC100279741	1.767649939
GRMZM2G052268	F-box domain containing protein	1.467482969
GRMZM2G054032	F-box protein	-2.725755655
GRMZM2G059799	Ethylene-responsive protein	2.515685089
GRMZM2G060257	ATPP2-A13	-1.035787149
GRMZM2G060276	hypothetical protein LOC100382494	1.259820284
GRMZM2G064954	Uncharacterized protein	1.616867462
GRMZM2G065970	unknown	1.467482969
GRMZM2G067460	Hypothetical protein LOC100381788	-1.121355175
GRMZM2G067626	hypothetical protein LOC100192470	-4.088633483
GRMZM2G068586	hypothetical protein LOC100193706	1.602251813
GRMZM2G069649	F-box family member	-1.128210122
GRMZM2G071113	hypothetical protein LOC100192671	1.510644232
GRMZM2G071705	cyclin-like F-box	7.337414843
GRMZM2G071997	Putative uncharacterized protein	1.605059497
GRMZM2G072028	hypothetical protein LOC100216622	2.641469343
GRMZM2G07	hypothetical protein LOC100274878	4.402448906

2586		
GRMZM2G074245	hypothetical protein LOC100277288	-2.306408631
GRMZM2G078198	Putative uncharacterized protein Transferase, transferring glycosyl groups	1.448669084
GRMZM2G079031	unknown	1.164203155
GRMZM2G081032	unknown	2.934965937
GRMZM2G081172	hypothetical protein LOC100275642	1.222902474
GRMZM2G082346	hypothetical protein LOC100276415	2.934965937
GRMZM2G082853	hypothetical protein LOC100381683	1.177043631
GRMZM2G082964	F-box domain containing protein	-1.362877828
GRMZM2G084035	F-box domain containing protein	1.065672156
GRMZM2G088482	ATPP2-A13	-13.62877828
GRMZM2G090104	Putative uncharacterized protein	1.027238078
GRMZM2G091293	hypothetical protein LOC100384571	1.254461247
GRMZM2G100121	unknown	1.076154177
GRMZM2G101036	F-box domain containing protein	3.179546432
GRMZM2G101060	Putative uncharacterized protein	-1.253847601
GRMZM2G101545	hypothetical protein LOC100193191	3.668707421

GRMZM2G102189	kelch motif family protein	-1.312400871
GRMZM2G104882	Hypothetical protein LOC100194140	1.956643958
GRMZM2G106363	hypothetical protein LOC100279726	-3.469143561
GRMZM2G107945	hypothetical protein LOC100383277	-1.066600039
GRMZM2G108228	LOC100281288	1.070039665
GRMZM2G109140	ubiquitin-protein ligase	-2.33636199
GRMZM2G110057	unknown	-1.55757466
GRMZM2G110330	unknown	-1.362877828
GRMZM2G113244	hypothetical protein LOC100276321	2.040718503
GRMZM2G115701	hypothetical protein LOC100273859	4.6470294
GRMZM2G115998	hypothetical protein LOC100194199	1.699190806
GRMZM2G116603	F-box domain containing protein	-2.385036198
GRMZM2G117633	Putative uncharacterized protein	-1.267237278
GRMZM2G118109	hypothetical protein LOC100194002	26.41469343
GRMZM2G119650	F-box domain containing protein Putative uncharacterized protein	1.731629903
GRMZM2G119932	Uncharacterized protein	-2.141665158
GRMZM2G12	F-box family protein Fragment	4.483975737

0408		
GRMZM2G123128	lysM domain containing protein	-1.734571781
GRMZM2G125411	hypothetical protein LOC100382859	-2.964259275
GRMZM2G126920	hypothetical protein LOC100384433	-1.362877828
GRMZM2G128215	F-box protein interaction domain containing protein	-1.362877828
GRMZM2G129288	Hypothetical protein LOC100383825	-1.207120362
GRMZM2G130109	hypothetical protein LOC100193239	1.167996648
GRMZM2G132913	F-box domain containing protein	-1.362877828
GRMZM2G133021	hypothetical protein LOC100382330	1.280304018
GRMZM2G133428	hypothetical protein LOC100273107	-25.21323981
GRMZM2G133895	hypothetical protein LOC100383858	9.171768553
GRMZM2G134550	Putative uncharacterized protein	1.051331082
GRMZM2G135002	hypothetical protein LOC100273406	-1.146056355
GRMZM2G135978	hypothetical protein LOC100274630	1.868481222
GRMZM2G137029	Uncharacterized protein	1.510644232
GRMZM2G137451	hypothetical protein LOC100216731	1.343818673
GRMZM2G137582	LOC100281166	1.467482969

GRMZM2G139066	F-box domain containing protein	1.614231265
GRMZM2G141472	hypothetical protein LOC100274409	-1.362877828
GRMZM2G142043	Uncharacterized protein	1.231961011
GRMZM2G144615	hypothetical protein LOC100272343	-1.542481882
GRMZM2G146694	unknown	1.956643958
GRMZM2G147402	hypothetical protein LOC100193397	1.073930718
GRMZM2G147800	unknown	1.020567701
GRMZM2G149153	Uncharacterized protein	1.191978503
GRMZM2G150169	unknown	1.236490279
GRMZM2G151496	hypothetical protein LOC100382223	1.926071396
GRMZM2G152290	Putative uncharacterized protein	-1.548724804
GRMZM2G155849	transport inhibitor response 1 protein	-1.829317298
GRMZM2G156490	hypothetical protein LOC100382083	-1.567309502
GRMZM2G156506	hypothetical protein LOC100304291	-2.271463046
GRMZM2G157132	hypothetical protein LOC100191208	-1.943362828
GRMZM2G157631	F-box domain containing protein	-3.738179184
GRMZM2G16	Putative uncharacterized protein	1.39082341

3726		
GRMZM2G165007	hypothetical protein LOC100274214	2.538348919
GRMZM2G169227	F-box domain containing protein	2.934965937
GRMZM2G171022	F-box domain containing protein	2.934965937
GRMZM2G171713	6-phosphofructokinase complex	2.201224453
GRMZM2G176340	hypothetical protein LOC100191954	2.777735619
GRMZM2G176638	Uncharacterized protein	2.690385442
GRMZM2G179532	F-box domain containing protein Putative uncharacterized protein	1.274393104
GRMZM2G180458	Putative uncharacterized protein	-2.11161935
GRMZM2G305864	Lipid binding protein	6.918133994
GRMZM2G313041	unknown	-1.42482682
GRMZM2G317262	Uncharacterized protein	-2.725755655
GRMZM2G330526	Cyclin-like F-box	1.318017111
GRMZM2G360352	Uncharacterized protein	-1.294733936
GRMZM2G365677	F-box protein interaction domain containing protein	3.197016467
GRMZM2G377373	unknown	-1.362877828
GRMZM2G379804	unknown	1.19059939

	GRMZM2G405474	hypothetical protein LOC100274552	1.320734672
	GRMZM2G434518	Putative uncharacterized protein	2.005560057
	GRMZM2G435445	hypothetical protein LOC100273081	-1.879119429
	GRMZM2G442489	hypothetical protein LOC100273804	1.180366736
	GRMZM2G443889	unknown	1.834353711
	GRMZM2G447480	Uncharacterized protein	3.668707421
	GRMZM2G459166	F-box protein GID2	2.934965937
	GRMZM2G702889	Putative uncharacterized protein	-1.263258453
E3 ubiquitin ligases_U Box	GRMZM2G007486	ubiquitin ligase complex	1.074715468
	GRMZM2G018059	ubiquitin ligase complex	7.92440803
	GRMZM2G019777	Uncharacterized protein	-3.407194569
	GRMZM2G020196	Uncharacterized protein	-1.265529411
	GRMZM2G025037	Putative ARM repeat-containing protein containing family protein	1.001991059
	GRMZM2G025214	STIP1 homology and U box-containing protein 1	1.19059939
	GRMZM2G027375	unknown	-7.268681747
	GRMZM2G030805	LOC100276970	1.195288547
	GRMZM2G03	LOC100273990	2.013886202

3521		
GRMZM2G03 7698	pre-mRNA-splicing factor 19	2.48223183
GRMZM2G05 0734	LOC100281409	1.570464229
GRMZM2G05 5052	Putative uncharacterized protein	1.222902474
GRMZM2G05 7436	Putative ARM repeat-containing protein containing family protein isoform 1	1.539823678
GRMZM2G05 9042	photoperiod responsive protein	-3.270906786
GRMZM2G06 3394	Uncharacterized protein	1.124029508
GRMZM2G06 5612	ubiquitin-protein ligase	3.668707421
GRMZM2G07 3310	Uncharacterized protein	1.781943605
GRMZM2G07 5104	Uncharacterized protein	1.385956137
GRMZM2G09 2550	unknown	3.179546432
GRMZM2G10 0090	ubiquitin-protein ligase	-1.817170437
GRMZM2G10 4769	Putative uncharacterized protein STIP1 homology and U box-containing protein 1	1.895498834
GRMZM2G11 5000	hypothetical protein LOC100383373	1.237289562
GRMZM2G12 5034	Uncharacterized protein	3.668707421
GRMZM2G12 7690	Uncharacterized protein	-4.088633483
GRMZM2G15 2857	ubiquitin ligase complex	-1.49916561

	GRMZM2G152919	hypothetical protein LOC100383857	1.877013099
	GRMZM2G160370	hypothetical protein LOC100381946	3.410906359
	GRMZM2G303964	U-box domain containing protein	-5.45151131
	GRMZM2G304010	Putative RING finger and ARM repeat-containing protein containing family protein	1.385956137
	GRMZM2G305822	Putative U-box domain protein kinase family	-1.728835763
	GRMZM2G307567	Uncharacterized protein	-1.109665554
	GRMZM2G314412	unknown	-4.770072396
	GRMZM2G324540	hypothetical protein LOC100191855	1.880858453
	GRMZM2G376085	unknown	-1.124374208
	GRMZM2G389462	unknown	1.687605414
	GRMZM2G425965	LOC100281122	-3.407194569
	GRMZM2G426271	Hypothetical protein LOC100193745	3.268484794
	GRMZM2G433433	Putative U-box domain protein kinase family	9.538639295
	GRMZM2G452016	hypothetical protein LOC100279362	-1.158446153
	GRMZM2G471733	hypothetical protein LOC100193248	3.301836679
	GRMZM2G476914	ubiquitin-protein ligase	2.65981288
E3 ubiquitin ligases_DDB	AC149475.2_F	proteasome subunit alpha type 5	1.962609336

G003		
GRMZM2G005080	Proteasome subunit alpha type (EC 3.4.25.1)	-1.86821455
GRMZM2G016511	maize 20S proteasome alpha subunit	1.612211793
GRMZM2G027282	hypothetical protein LOC100273594	1.047923343
GRMZM2G029583	26S proteasome non-ATPase regulatory subunit 6	1.928302012
GRMZM2G033626	26S proteasome non-ATPase regulatory subunit 14	1.586790527
GRMZM2G038126	26S proteasome regulatory complex ATPase RPT3	1.109210759
GRMZM2G051790	hypothetical protein LOC100273813	2.736657428
GRMZM2G056569	Putative uncharacterized protein	1.042449792
GRMZM2G056870	proteasome subunit alpha type 5	2.695589283
GRMZM2G061745	hypothetical protein LOC100274928	1.119071132
GRMZM2G074037	Hypothetical protein LOC100273945	1.247360523
GRMZM2G074549	hypothetical protein LOC100216834	1.453725316
GRMZM2G090904	26S proteasome regulatory complex ATPase RPT3	2.965538499
GRMZM2G092975	hypothetical protein LOC100274112	1.486541189
GRMZM2G093557	LOC100282612	2.06781691
GRMZM2G104373	hypothetical protein LOC100279843	1.536703863

	GRMZM2G107362	hypothetical protein LOC100274445	1.37019128
	GRMZM2G117544	hypothetical protein LOC100272320	-1.33896769
	GRMZM2G120047	hypothetical protein LOC100191947	-1.194851794
	GRMZM2G126956	Hypothetical protein LOC100381699	3.118401308
	GRMZM2G137528	hypothetical protein LOC100273032	1.232207167
	GRMZM2G140867	LOC100283485	1.251489804
	GRMZM2G171604	26S protease regulatory subunit S10B	1.84319397
	GRMZM2G181359	hypothetical protein LOC100272769	1.284047597
	GRMZM2G368908	hypothetical protein LOC100276453	-1.037787704
	GRMZM2G402002	hypothetical protein LOC100279448	1.429530823
	GRMZM2G467059	26S proteasome non-ATPase regulatory subunit 14	1.463241688
26S proteasome	AC149475.2_FG003	proteasome subunit alpha type 5	1.962609336
	GRMZM2G005080	hypothetical protein LOC100193140	-1.86821455
	GRMZM2G016511	maize 20S proteasome alpha subunit	1.612211793
	GRMZM2G027282	26S protease regulatory subunit 6A	1.047923343
	GRMZM2G029583	26S proteasome non-ATPase regulatory subunit 6	1.928302012
	GRMZM2G03	26S proteasome non-ATPase regulatory subunit 14	1.586790527

3626		
GRMZM2G038126	26S proteasome regulatory complex ATPase RPT3	1.109210759
GRMZM2G056569	Uncharacterized protein	1.042449792
GRMZM2G056870	proteasome subunit alpha type 5	2.695589283
GRMZM2G061745	hypothetical protein LOC100274928	1.119071132
GRMZM2G074037	Putative uncharacterized protein	1.247360523
GRMZM2G074549	hypothetical protein LOC100216834	1.453725316
GRMZM2G090904	26S proteasome regulatory complex ATPase RPT3	2.965538499
GRMZM2G092975	hypothetical protein LOC100274112	1.486541189
GRMZM2G093557	26S proteasome non-ATPase regulatory subunit 6 Putative uncharacterized protein	2.06781691
GRMZM2G104373	26S protease regulatory subunit 4	1.536703863
GRMZM2G107362	26S protease regulatory subunit 8 Putative uncharacterized protein	1.37019128
GRMZM2G117544	hypothetical protein LOC100272320	-1.33896769
GRMZM2G120047	proteasome core complex, alpha-subunit complex	-1.194851794
GRMZM2G137528	hypothetical protein LOC100273032	1.232207167
GRMZM2G140867	LOC100283485	1.251489804
GRMZM2G171604	26S protease regulatory subunit S10B	1.84319397

	GRMZM2G181359	hypothetical protein LOC100272769	1.284047597
	GRMZM2G368908	hypothetical protein LOC100276453	-1.037787704
	GRMZM2G467059	26S proteasome non-ATPase regulatory subunit 14	1.463241688
Plant cysteine oxidases	GRMZM2G038677	hypothetical protein LOC100193573	9.957920143
	GRMZM2G043417	unknown	4.695945499
	GRMZM2G107557	Putative uncharacterized protein	18.408279
	GRMZM2G113355	hypothetical protein LOC100191278	2.308400175
	GRMZM2G136250	hypothetical protein LOC100191514	1.21591446
	GRMZM2G162175	Cupin, RmlC-type Putative uncharacterized protein	1.935559433

Table S8. Primer sequences of waterlogging responsive genes identified in various pathways in RNASeq for qRT-PCR validation

Gene ID	Annotation	Primer	Primer sequence	Tm	Length	GC %	Amplicon length
GRMZM2G136139	Neutral/alkaline invertase	F	ACCCGAGTTCTGGTTCTTTG	62	20	50	87
		R	AACATTGGACACGCCTACTT	62	20	45	
GRMZM2G159587	Glyoxylate reductase	F	CCAAGTCCAACCTCCCTAAAC	62	21	52.4	116
		R	CCAAGTCCAACCTCCCTAAAC	62	21	52.4	
GRMZM2G046686	Hexokinase-1	F	CGGTCTCTACCAAGGCTATTC	61	21	52.4	111
		R	CGGTCTCTACCAAGGCTATTC	61	22	50	
GRMZM2G119689	Beta-fructofuranosidase, insoluble isoenzyme 2	F	CGTGTTCTTCAGGGTGTTCA	62	20	50	137
		R	AGATGTCCGTGTCGACAAATC	62	21	47.6	
GRMZM2G430807	metallothionein-like protein type 2	F	CAGCAAAGCCCTGTAGTGATA	62	21	47.6	108
		R	AAGAGCTCTCTCACTCTCTCTC	62	22	50	
GRMZM2G402564	Metallothionein2	F	CTAGTGGAGGGAGTTGTCTAGT	62	22	50	121
		R	ACGATACCACGATGCAGATG	62	20	50	
GRMZM2G034896	hypothetical protein LOC100384248	F	CACAGACACAGTTGGGAAAGA	62	21	47.6	103
		R	CCAACTTTCGCAGTCACAAAC	62	21	47.6	
GRMZM2G036134	hypothetical protein LOC100191400	F	CTTTCGCAGACCAAGGACAT	62	20	50	84
		R	GAGCGTTCCAGAGAAGTTGAG	62	21	52.4	
GRMZM2G026855	hypothetical protein LOC100273291	F	GGGTTCATCCAGAGCGATTAC	62	21	52.4	104
		R	ACGCTCATGTCTTCCACATAC	62	21	47.6	
GRMZM2G119494	Glycoside hydrolase, family 28 Putative uncharacterized protein	F	ACCATGAAGTGGGTGTTCTG	62	20	50	109
		R	ACACGTCGCTGTAGTTGATG	62	20	50	
GRMZM2G342246	product:beta-expansin 7	F	CGTTCCTTCGATTCACTTCCT	62	21	47.6	102
		R	TTAGTAGGCCGACAGGAGAA	62	20	50	
GRMZM2G094523	Putative uncharacterized protein	F	GCGTTCCATCGACATCTCTAAT	62	22	45.5	122

		R	TGTGAACTGGTAAAGAGCAGAA	62	22	40.9	
AC177897.2_FG002	hypothetical protein LOC100274874	F	GAGCACAGCCACTACTAACAA	62	21	47.6	102
		R	GCGCATAAGGGTTGAACAAAG	62	21	47.6	
GRMZM2G118109	hypothetical protein LOC100194002	F	CTGAGGTGAGGTGTTTCCTAAA	62	22	45.4	98
		R	TGAGAGTCGTTGCCTCTACTA	62	21	47.6	
GRMZM2G340444	Putative RING zinc finger domain superfamily protein	F	TAACCAGCTCAGCGCTATTG	62	20	50	103
		R	TGAGGAAAGCGGAAGAAAGG	62	20	50	
GRMZM2G027120	protein binding protein (LOC100283571), mRNA	F	GGGAGAATGCATGTTCTGACT	62	21	47.6	97
		R	CAGCCAAAGGACGCAAATAAG	62	21	47.6	

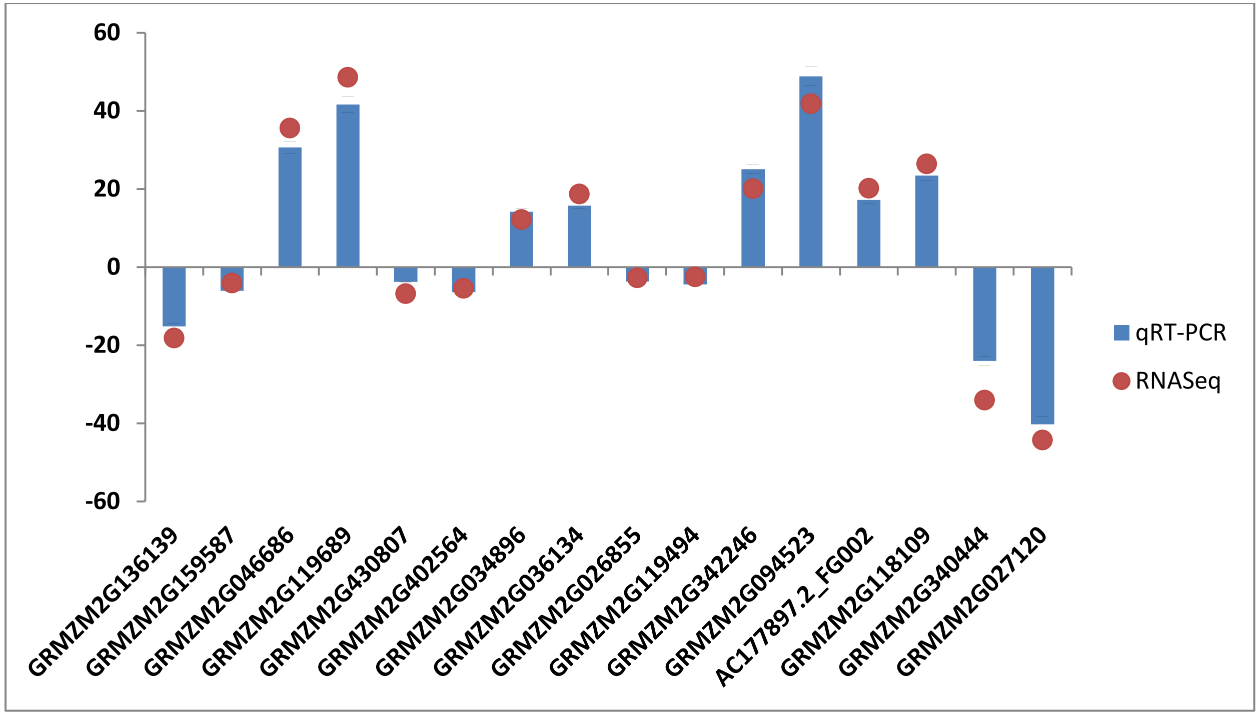

Supplementary Figure S1: Validation of selected genes operating in different pathways expressed in RNAseq by qRT-PCR. X-axis represents the gene models and Y-axis represents the log-transformed fold change expression values of genes. Error bar in the column represents the standard error.
